# Effects of gut microbiota dysbiosis on the metabolism and pharmacokinetics of losartan in rats: from endogenous to ceftriaxone-induced dysbiosis

**DOI:** 10.3389/fmicb.2025.1693247

**Published:** 2025-12-02

**Authors:** Jiaxuan Xia, Yibao Jin, Yanjun Hong, Yuefeng Zhang, Meifang Li, Houshuang Huang, Xu Cai, Dan Li, Bing Wang, Zhiyong Xie

**Affiliations:** 1 NMPA Key Laboratory for Bioequivalence Research of Generic Drug Evaluation, Shenzhen Institute for Drug Control, Shenzhen, China; 2 School of Pharmaceutical Sciences, Shenzhen Campus of Sun Yat-sen University, Shenzhen, China; 3 Department of Pharmacy, Renmin Hospital of Wuhan University, Wuhan, China

**Keywords:** gut microbiota dysbiosis, losartan potassium, pharmacokinetics, ceftriaxone sodium, *Enterococcus faecalis*

## Abstract

**Introduction:**

Gut microbiota plays a key role in drug metabolism. While gut microbiota dysbiosis is known to contribute to hypertension pathogenesis, its impact on drug metabolism remains poorly considered. Clinically, the pharmacokinetic variability of losartan potassium is partially attributed to genetic polymorphisms of the CYP2C9 enzyme and structural variations in AGTR1. However, the potential role of gut microbiota dysbiosis in regulating losartan pharmacokinetics remains unclear.

**Methods:**

In the present study, we assessed the effect of gut microbiota dysbiosis on the metabolism and pharmacokinetics of losartan in two different rat models: spontaneously hypertensive rats (SHRs) with endogenous gut microbiota dysbiosis and rats with ceftriaxone (CRO)-induced gut microbiota dysbiosis combined with *in vitro* and *in vivo* studies.

**Results:**

The results showed that the intestinal flora from SHRs led to a more significant degradation of losartan than that from Wistar Kyoto rats (WKYs) *in vitro*. More importantly, we observed a reduction in the oral bioavailability of losartan in rats with gut microbiota dysbiosis. Specifically, compared to WKYs, the AUC_0-∞_ of losartan and its active metabolite E-3174 decreased by 50.24% (*p* < 0.05) and 72.42% (*p* < 0.01), respectively, in SHRs. In the WKY + CRO group, losartan AUC_0-t_ decreased by 25.90% (*p* < 0.05) compared to WKYs; while the SHR + CRO group showed a 57.20% (*p* < 0.01) reduction compared to SHRs. Spearman correlation analysis of 16S rRNA full-length sequencing and pharmacokinetic parameters showed a significant negative correlation between *Enterococcus faecalis* (*E. faecalis*) abundance and losartan’s AUC_0-t_ and *C_max_*. *In vitro* experiments concluded that *E. faecalis* metabolized losartan and converted it into E-3179. The reduced oral bioavailability of losartan in rats with CRO-induced dysbiosis was likely due to the effect of *E. faecalis* in degrading losartan.

**Conclusion:**

This study highlights that gut microbiota dysbiosis diminishes losartan bioavailability, providing evidence that gut microbiota contributes to the pharmacokinetic variability of losartan.

## 1 Introduction

Hypertension is a significant contributor to mortality (Choi et al., 2024) and leads to various complications that severely impact individuals’ quality of life (Kario et al., 2024). The human body and animals are colonized by trillions of microbes, which are collectively referred to as the “microbiota” (Mishima and Abe, 2022). Under physiological conditions, there is a symbiotic balance between the gut microbiota and the host. The disruption of this balance, known as dysbiosis, has been implicated in a variety of pathological conditions, including hypertension (Avery et al., 2021). Evidence has shown that gut microbiota dysbiosis is observed both in individuals with high blood pressure patients (Li et al., 2017) and spontaneously hypertensive rats (SHRs) (Yang et al., 2015). Fecal microbiota transplantation (FMT) studies have demonstrated that the dysregulation of intestinal flora can aggravate the progression of hypertension (Adnan et al., 2017; Toral et al., 2019), while conventionalizing germ-free rats can alleviate hypertension (Joe et al., 2020). These findings collectively highlighted the importance of maintaining a balanced gut microbiota in managing hypertension.

In fact, the gut microbiome affects not only the body’s physiology and pathology but also how the body handles foreign substances, including oral antihypertension drugs (Qin et al., 2010). Recently studies have shown that approximately 2/3 of chemical drugs are metabolized by at least one human gut bacterium (Zimmermann et al., 2019). The gut microbiota is involved in drug metabolism, influencing it during enterohepatic circulation either pre-absorption or via gut microbial enzymatic reactions (Koppel et al., 2017). In addition, some drugs are metabolized by the intestinal microbiota into specific metabolites that cannot be formed in the liver. Furthermore, metabolizing drugs through the gut microbiota prior to absorption can alter the systemic bioavailability of certain drugs (Zhang et al., 2018). For example, a study has observed that the bioavailability of nifedipine was lower in SHRs than in Wistar rats, which is partly attributed to microbial biotransformation (Zhou et al., 2023). Pharmacokinetic analyses showed that systemic exposure of amlodipine was significantly elevated in antibiotic-treated rats compared with controls (Yoo et al., 2016). In this aspect, gut microbiota, one of the determinants of pharmacokinetics, has long been underestimated (Chen et al., 2021; Flowers et al., 2020; Tuteja and Ferguson, 2019; Dong et al., 2022).

Losartan potassium, as a first generation of Angiotensin II Receptor Blocker (ARB), have been widely used in the treatment of hypertension (Xu et al., 2009; Lee et al., 2023). After oral administration, losartan is first metabolized by the liver into E-3179, an inactive metabolite (Krämer et al., 2002), and then further into E-3174 (Goa and Wagstaff, 1996), which is a more potent Angiotensin II Type 1 Receptor (AGTR1) blocker. However, only about 14% of losartan is converted into E-3174. Notably, after administration, approximately 35% of losartan is excreted in urine and 60% in feces (Zhou et al., 2009; Mukai et al., 2015), this significant gut exposure provides opportunities for interactions with the gut microbiota. A clinical pharmacokinetic study of losartan potassium (10–150 mg) in patients with mild-to-moderate essential hypertension showed consistent safety but variable tolerance, indicating pharmacokinetic heterogeneity (Gradman et al., 1995). While CYP2C9 gene polymorphisms (Park et al., 2021) and AGTR1 receptor structural variations (Zeng et al., 2023) are known contributors to this heterogeneity, other factors cannot be ruled out. Additionally, an *in vitro* investigation indicated that losartan can be degraded by approximately 20% when exposed to fecal samples from healthy individuals (Zimmermann et al., 2019). The microbiome’s ability to process various drugs is well-known. However, the extent to which it impacts in drug pharmacokinetics remains uncertain. In particular, it is unclear whether the gut microbiota contributes to variability in the clinical pharmacokinetics of drugs, and if so, to what extent.

With the advancement of sequencing technologies, techniques such as microbiome-derived metabolites screening (Javdan et al., 2020) and high-throughput anaerobic screening (Müller et al., 2024) for identifying compounds acting against gut bacteria in monocultures or communities have enabled the identification of numerous bacterial species with bioconversion activity against various drugs (Viglioli et al., 2024; Gu et al., 2025). Moreover, altering the gut microbiota through different means was used to evaluate its impact on drug metabolism *in vivo* (Verdegaal and Goodman, 2024; Alam et al., 2025). For example, an antibiotic cocktail was used to study the influence of gut microbiota on the pharmacokinetics of nifedipine in SHRs (Zhou et al., 2023). While in the FMT, the purpose of the antibiotic treatment was to reduce the existing gut microbiota and facilitate the establishment of microbial populations and diversity from donor rats after FMT (Li et al., 2017). Recently, ceftriaxone (CRO) has been used as gut microbiota dysbiosis inducers to study its impact on dysbiosis and changes in intestinal structure in adjuvant obesity treatment (Aparecida Dos Reis Louzano et al., 2022). Considering the clinical relevance of the gut microbiota to antihypertensive drugs, the present study aimed to investigate gut microbiota dysbiosis (from endogenous dysbiosis to CRO-induced dysbiosis) and explore the effects of intestinal flora on the metabolism and pharmacokinetics of losartan potassium. This study will contribute to elucidating the relationship between gut microbiota dysbiosis and the metabolism of losartan, providing a basis for personalized antihypertensive therapy targeting microbiota-drug interactions.

## 2 Materials and methods

### 2.1 Chemicals and reagents

Losartan potassium reference substance (B100597-202104, 99.8%) was provided by the National Institutes for Food and Drug Control (Beijing, China). Authentic standards for E-3174 (E286809), irbesartan (I129263) (internal standard, IS), and heparin sodium salt (H123383-250KU) were purchased from Aladin (Shanghai, China). E-3179 (L905068) and ceftriaxone disodium salt hemiheptahydrate (C832409) were obtained from Macklin (Shanghai, China). L-cysteine (E200-158-2) was from Sangon Biotech (Shanghai, China). Gifu Anaerobic Medium modified (HB8518-3), vitamin K1 (2100501), and Hemin Chloride (2100500) were purchased from Qingdao Hi-tech Industrial Park Hope Bio-technology Co., Ltd. HPLC grade acetonitrile was from Fisher Scientific (Pittsburgh, PA, United States). MS grade acetic acid and formic acid were from Aladin (Shanghai, China). Ultrapure water for liquid chromatography coupled with mass spectrometry (LC–MS) analysis was supplied by a Millipore Milli-Q water purification system (Bedford, MA, United States).

### 2.2 Animals and experimental design

Male 16-week-old Wistar Kyoto (WKY) rats and SHRs weighing about 260–300 g were purchased from the Beijing Vital River Laboratory Animal Technology Co., Ltd. (Beijing, China). The certificate was SCXK (JING) 2021–0253. All animals were kept on a 12 h light and dark cycle, with a temperature of 21–24 °C, and humidity of 40–60%, with free access to laboratory animal maintenance feed and water (Shenzhen Institute for Drug Control, Shenzhen, China). Blood pressure was measured after 1 week of adaptive feeding, while systolic blood pressure (SBP) > 150 mmHg of SHR was available for the study. Feces, serum, and urine samples were collected and stored at −80 °C until analysis. The experiment was approved by the Ethics Committee of Shenzhen Institute for Drug Control (Approval number: SZIDC-YL-20230904).

### 2.3 Fecal collection, processing and incubation with the drug

Fresh fecal samples from WKYs and SHRs were collected and immediately transferred to an anaerobic chamber (90% N₂, 5% CO₂, 5% H₂). Fecal suspensions were prepared by homogenizing 1 g of feces in 15 mL of sterile phosphate-buffered saline containing 0.1% L-cysteine. After 5 min of sedimentation of insoluble particles, supernatants were collected and inoculated into modified GAM broth (mGAM) (Javdan et al., 2020). Cultures were anaerobically incubated at 37 °C for 24 h to enrich gut microbiota. For drug metabolism assays, a fixed concentration of 40 μM was used (Maier et al., 2018). Bacteria-free controls contained equivalent drug volumes consistently. All conditions were incubated anaerobically at 37 °C for 12, 24, and 48 h, with triplicate samples per condition. Drug remaining content and non-target metabolites screen were detected by ultra-high performance liquid chromatography coupled with triple quadrupole mass spectrometry (UHPLC-TQ-MS) and ultra-high performance liquid chromatography with quadrupole time-of-flight mass spectrometry (UHPLC-Q-TOF-MS), respectively. The sample preparation process can be found in the Supplementary material. For UHPLC-Q-TOF-MS analysis, the MS parameters and IDA criteria can be seen in Supplementary Table S1.

### 2.4 Pharmacokinetic experiments

The pharmacokinetic study was described as follows. After fasting overnight, 20 mg/kg (Robles-Vera et al., 2020) of losartan potassium (water as solvent) was administered to the WKYs and SHRs by intragastric gavage. Blood samples were taken from the rats at 0, 0.25, 0.5, 0.75, 1, 2, 3, 4, 6, 8, 10, 12, and 24 h (Cusinato et al., 2019). The blood samples were centrifuged at 4,000 rpm at 4 °C for 20 min to isolate plasma for determining the concentration of losartan and its metabolites. After the first pharmacokinetic study, a two-day recovery period was allowed before inducing gut dysbiosis with ceftriaxone sodium. Ceftriaxone sodium was administered to induce gut dysbiosis as follows: rats were given ceftriaxone sodium (400 mg/kg/day) orally once daily for the first week, followed by administration every 2 days for the subsequent week, totaling 2 weeks, according to a protocol described in a previous study with minor modifications (Almugadam et al., 2021). After another two-day recovery period, the second pharmacokinetic study was performed on the same batch of animals treated with ceftriaxone sodium, following the same protocol as the first.

**Figure 1:**
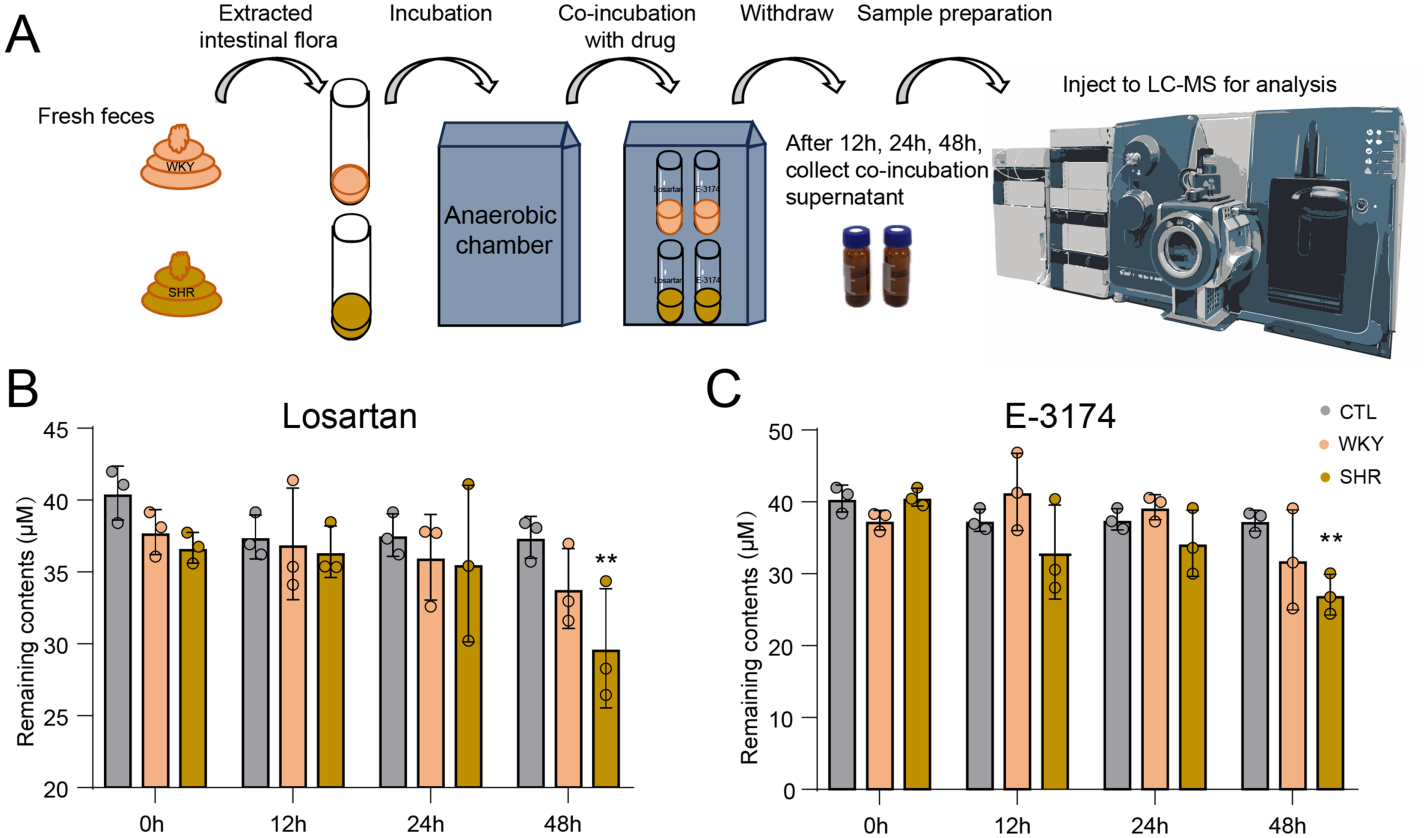
Drug-metabolizing activities of rat gut bacteria. **(A)** Schematic of the drug incubation assay. **(B)** Remaining content of losartan and **(C)** E-3174 after incubation with rat fecal suspension at various time points. Data are expressed as mean ± SD, ^**^p < 0.01.

### 2.5 Metabolites detection *in vivo*

After a single oral dose of losartan potassium (20 mg/kg) was administered to the WKYs and SHRs, respectively, biological samples were collected as follows: Blood samples were drawn from the orbital venous plexus pre-dose (0 h) and at 0.5, 1, 2, 3, 4, 6, 8, and 12 h post-dosing. The blood samples were centrifuged at 4,000 rpm at 4 °C for 20 min to isolate plasma. For urine and feces, cumulative samples were collected over the following time intervals: 0–4, 4–8, 8–12, and 12–24 h after administration. The processing of the biological samples can be found in the Supplementary material.

### 2.6 Bacteria strains, cultivate and co-inoculate with the drug

*Enterococcus faecalis* (ATCC 51299, BNCC) was cultured in modified brain heart infusion (BHI) broth supplemented with L-cysteine HCl (0.5 g/L), vitamin K1 (10 mg/L), hemin (5 mg/L) and resazurin (1 mg/L). *Limosilactobacillus reuteri* (JCM 1112, Guangdong Academy of Sciences) was cultured in MRS medium at 37 °C in an anaerobic chamber (5%H_2_, 5%CO_2_ and 90%N_2_). Drug metabolism assays were performed in 96-well plates under optimal growth conditions for each strain. The strains were inoculated at a 1:100 (v/v) ratio into media containing either losartan potassium or E-3174. Each experiment comprised three biological replicates, including a bacteria-free control. The growth of strains was monitored by measuring OD_600_ (Eon Microplate Spectrophotometer, BioTek) every 2 h for the first 10 h, followed by 12 to 24 h intervals until 72 h. Supernatants were withdrawn at 6, 12, 24, 48, and 72 h. The remaining drug content and the formation of E-3179 were quantified via UHPLC-TQ-MS. The sample preparation for quantitative analysis was the same as in section 2.3.

### 2.7 LC–MS/MS method

The UHPLC-TQ-MS analysis was conducted on an LC-30 AD ultra high-performance liquid chromatograph (Shimadzu, Japan) connected to an AB 4500 (AB Sciex, United States) Triple Quadrupole Mass Spectrometer with an ESI source. Chromatographic separation was achieved on an Acquity UHPLC HSS T3 column (100 × 2.1 mm, 1.8 μm) at 40 °C, and the eluent was aqueous formic acid (100:0.1, v/v) (A) and acetonitrile with formic acid (100:0.1, v/v) (B) at a flow rate of 0.3 mL min^−1^. Gradient elution differs in the calculation of residual content and pharmacokinetic experiments. MRM mode was employed. Data processing was performed using Analyst Software (AB Sciex). The UHPLC-TQ-MS method validation was conducted in terms of selectivity, linearity, carryover, accuracy and precision, matrix effect, extraction recovery, dilution integrity, and stability under various conditions, which was performed in line with the FDA guidelines for bioanalytical assays. More details were described in the Supplementary material method.

### 2.8 Bacterial quantification

Fecal samples were freshly collected, rapidly frozen, and weighed before storage at −80 °C until use. The absolute quantification of bacterial 16S rRNA amplicon sequencing was conducted by Majorbio Bio-Pharm Technology Co. Ltd. in Shanghai, China. Total microbial genomic DNA was extracted using the E. Z. N. A.^®^ Soil DNA Kit, following the manufacturer’s guidelines. The quality and concentration of the extracted DNA were assessed through 1.0% agarose gel electrophoresis and with a NanoDrop 2000 spectrophotometer. RT-PCR was performed using universal 16S primers (Forward: 5′-TCCTACGGGAGGCAGCAGT-3′, Reverse: 5′-GGACTACCAGGGTATCTAATCCTGTT-3′) (Nadkarni et al., 2002). The relative bacterial load was determined by normalizing the DNA content against the weight of feces from the untreated group.

### 2.9 Gut microbiota analysis

Fecal DNA was extracted utilizing the E. Z. N. A.® Soil DNA Kit. The extracted DNA was analyzed using a 1% agarose gel, and its concentration and purity were assessed with NanoDrop2000 spectrophotometer. For 16S rRNA gene sequencing, universal primers of the V3-V4 region, 338F (5′-ACTCCTACGGGAGGCAGCAG-3′) and 806R (5′-GGACTACHVGGGTWTCTAAT-3′) (Liu et al., 2016), were employed in conjunction with the fecal DNA and fluorescent dye for PCR amplification. For 16S rRNA full-length sequencing, the primers 27F (5′-AGRGTTYGATYMTGGCTCAG-3′) and 1492R (5′-RGYTACCTTGTTACGACTT-3′) were used. The resulting PCR products were purified, quantified, and homogenized to create a sequencing library, which was commissioned to Majorbio Bio Pharm Technology Co. Ltd. The sequencing data were processed using the DADA2 algorithm in Qiime 2 to obtain amplicon sequence variants (ASVs), followed by the removal of chloroplast and mitochondrial sequences. Samples were standardized to sequencing depth. To minimize the effects of sequencing depth on alpha and beta diversity measure, the number of sequences from each sample was rarefied. Taxonomic assignment of ASVs was performed using the Naive Bayes consensus taxonomy classifier implemented in Qiime2 and the SILVA 16S rRNA database (v138). Based on the ASVs information, rarefaction curves and alpha diversity indices were calculated with Mothur v1.30.1. Rarefaction curves were produced for individual samples to evaluate the depth of sequencing. Bioinformatic analysis of the 16S/ PK parameters was carried out using the Majorbio Cloud platform.

For microbial community analysis, alpha diversity indices such as Chao1 and Shannon index were included, and the Wilcoxon rank-sum test was used to analyze the differences among groups. The similarity of microbial community structure among samples was evaluated by Principal Coordinates Analysis (PCoA) based on Bray-Curtis distance, and the significance of differences in microbial community structure among groups was assessed using PERMANOVA. To predict distinct species in gut microbiota dysbiosis in rats induced by CRO, random forest algorithms were used to identify “treated” and “untreated” CRO rats based on the relative abundance of selected bacterial genera. Line discriminant analysis effect size (LEfSe) was used to evaluate the effect size of differential features (i.e., LDA score). Random forest models were constructed using differential microbial genera (*p* < 0.05) identified by Wilcoxon rank-sum tests. The accuracy of the top 20 bacterial genera and pharmacokinetic parameters was then assessed individually and in combination by Area Under the ROC Curve (AUC) using the “ROC. curve” package in R. Bacteria with significant differences were selected based on the variable importance (VIP) in the key pharmacokinetic parameters, which were obtained from the orthogonal least partial squares discriminant analysis model. The selection criteria were a VIP value greater than 1 and a *p* value less than 0.05 from the Wilcoxon test. Species with a Spearman correlation coefficient *r* > 0.6 and *p* < 0.05 were selected for correlation network analysis to evaluate the relationships between different bacterial species and their associations with pharmacokinetic parameters.

### 2.10 Statistical analysis

The pharmacokinetic parameters of losartan, E-3174, and E-3179 were calculated using the non-compartment analysis model with Drug and Statistics (DAS) software (version 2.0). The comparisons of the relative abundance of the genera between different groups were performed using the Wilcoxon rank-sum test. Comparisons between WKY and SHRs before and after CRO treatment were conducted using either a *t*-test or one-way ANOVA, with *p* values < 0.05 considered statistically significant. All results were presented as mean ± standard deviation. Graphs were created using GraphPad Prism 8.0 software.

## 3 Results

### 3.1 Gut microbiota from SHRs significantly metabolizes losartan and E-3174 *in vitro*

To investigate the metabolic ability of gut microbiota on losartan and its active metabolite E-3174, *in vitro* culture incubation of the intestinal flora from WKYs and SHRs with these two drugs under anaerobic conditions was conducted, along with three vehicle controls without the drug or bacteria (Figure 1A). Drug concentrations were measured at 0, 12, 24, and 48 h using LC–MS/MS. Qualitative analysis revealed that neither losartan nor E-3174 was metabolized to other compounds by the gut microbiota in either WKY (Supplementary Figures S1A–D) or SHR (Supplementary Figures S1E–H) groups *in vitro*. However, quantitative analysis showed that gut microbiota from both groups were capable of degrading losartan and E-3174 to varying extents (Figures 1B,C; Supplementary Table S2). Notably, after 48 h of drug and bacteria co-incubation, the remaining contents of losartan and E-3174 were significantly reduced in SHRs when compared to WKYs.

**Figure 2:**
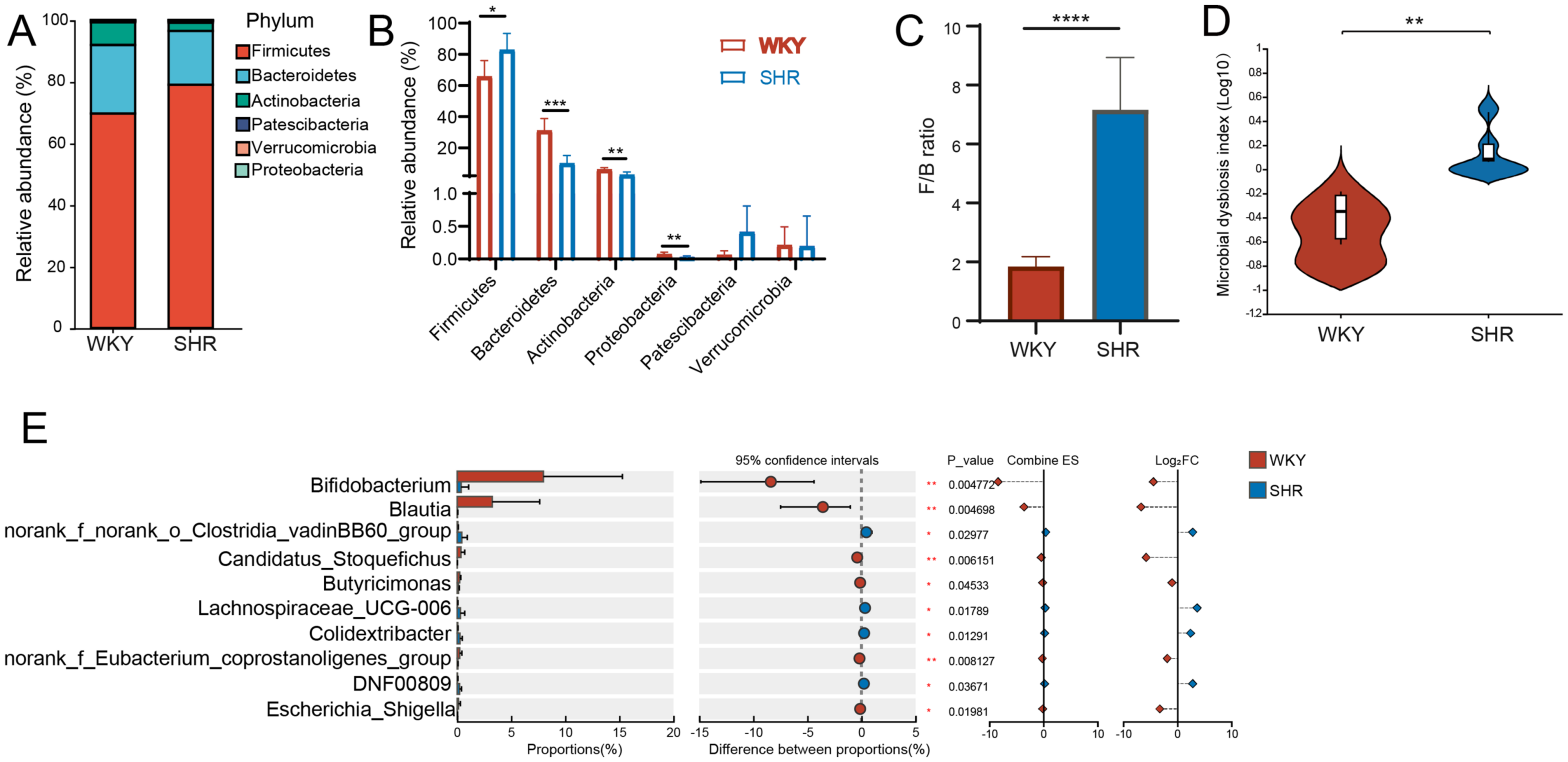
Gut microbial dysbiosis in SHRs. **(A)** Composition of the gut microbiome at phylum taxonomic levels; **(B)** Relative abundance of phyla in each group; **(C)** The value of the F/B ratio; **(D)** MDI index in each group; **(E)** Top 10 most abundant genera with content differences between the two groups. Data are expressed as mean ± SD. ^*^p < 0.05, ^**^p < 0.01, ^***^p < 0.001, ^****^p < 0.001, unpaired *t* test or Wilcoxon rank-sum test.

### 3.2 Lower abundance of losartan and its metabolites in plasma and feces in SHRs compared with WKYs

To comprehensively profile the metabolites of losartan potassium, a single oral gavage of losartan potassium was administered to WKYs and SHRs. The metabolites in plasma, feces, and urine samples were analyzed using nontargeted screening with UHPLC-Q-TOF-MS. In addition to the known metabolites E-3174 and E-3179, other metabolites with an [M + H]^+^ of 439.16 were detected in feces and plasma (Supplementary Figures S2A–D). These metabolites, which had four isomers, had higher mass spectral intensity in feces and plasma. Comparison of the fragments of these four isomers (m/z 439.16) with those of losartan revealed similar fragmentation patterns (Supplementary Figures S3A–E), indicating that these metabolites were derivatives of losartan. Previous studies have shown that losartan can be hydroxylated to an inactive metabolite involving modification of the butyl side chain (Stearns et al., 1992; Chen et al., 1993), which aligns with our findings. Notably, the abundance of losartan, E-3174, and E-3179, including the metabolites with m/z 439.16, was lower in SHRs than in WKYs in both feces and plasma, while losartan and E-3179 were more abundant in urine (Supplementary Figure S3F).

### 3.3 Decreased oral bioavailability of losartan and E-3174 and gut microbial dysbiosis in SHRs

We then conducted pharmacokinetic studies on WKYs and SHRs. A sensitive LC–MS/MS method was developed to quantify losartan, E-3174, and E-3179 in rat plasma, using irbesartan as the internal standard (IS). The validation results of the LC–MS/MS method demonstrated that it met the requirements for pharmacokinetic analysis (Supplementary Figure S4; Supplementary Tables S3–S6). The pharmacokinetic parameters of losartan, E-3174, and E-3179 were analyzed, including area under the concentration-time curve (AUC), maximum concentration (*C*_max_), mean residence time (MRT), elimination half-life (*T*_1/2_), and time to reach maximum concentration (*T*_max_). These analyses revealed marked differences in the pharmacokinetic characteristics of losartan, E-3174, and E-3179 between WKYs and SHRs. In the WKYs, the values of losartan in AUC_0–t_ and AUC_0-∞_ were 29942.21 ± 15428.31 ng·h/mL and 39156.13 ± 20370.82 ng·h/mL, respectively; while in the SHRs, it showed a significantly reduced AUC_0-t_ and AUC_0-∞_, declining by 39.88% (18002.60 ± 2806.38 ng·h/mL) and 50.24% (19483.83 ± 4098.26 ng·h/mL, *p* < 0.05), respectively (Table 1). Similar trends were observed in E-3174 and E-3179, which displayed 72.42% (*p* < 0.01) and 36.13% reductions in AUC_0-∞_, respectively. Furthermore, the *C*_max_ values of losartan, E-3174, and E-3179 in SHRs showed a diminished trend compared to those in WKYs. However, the other pharmacokinetic parameters, such as MRT and *T*_1/2_, showed no significant difference in the WKY and SHR groups (Supplementary Table S7).

**Table 1:** Pharmacokinetic parameters of losartan, E-3174 and E-3179 in WKYs and SHRs.

Analytes	Parameter	WKY	SHR	WKY + CRO	SHR + CRO
Losartan	AUC_0-t_ (ng.h/mL)	29942.21 ± 15428.31	18002.60 ± 2806.38	22185.17 ± 16026.77^#^	7702.83 ± 1695.54^##^
	AUC_0-∞_ (ng.h/mL)	39156.13 ± 20370.82	19483.83 ± 4098.26*	43978.196 ± 50647.491	8628.79 ± 2873.97^##^
	C_max_ (ng/mL)	9331.32 ± 7242.24	4004.48 ± 1771.38	2017.49 ± 991.45^#^	2026.206 ± 912.90
E-3174	AUC_0-t_ (ng.h/mL)	64964.25 ± 44071.53	17606.01 ± 4274.64*	36486.97 ± 19882.15	13513.88 ± 4944.11
	AUC_0-∞_ (ng.h/mL)	78303.78 ± 41083.80	21598.97 ± 3067.90**	96286.31 ± 45590.98	18239.82 ± 11548.85
	C_max_ (ng/mL)	6985.09 ± 6023.88	1801.94 ± 1113.59	2293.72 ± 1317.04	1083.05 ± 513.08
E-3179	AUC_0-t_ (ng.h/mL)	1336.14 ± 740.83	850.57 ± 189.22	1125.744 ± 880.79	339.46 ± 76.76^##^
	AUC_0-∞_ (ng.h/mL)	1410.02 ± 741.09	900.54 ± 226.29	2052.634 ± 3057.82	362.64 ± 105.56^##^
	C_max_ (ng/mL)	310.47 ± 243.53	143.43 ± 108.27	80.24 ± 43.24^#^	43.54 ± 12.16

The data are expressed as the mean ± standard deviation (SD), *n* = 6 group. “*” means WKY vs SHR. “^#^” means WKY vs WKY+CRO and SHR vs SHR+ABX. * or ^#^ means *p* < 0.05, **or ^##^ means *p* < 0.01.

Considering the relationship between gut microbiota and hypertension, we investigated the changes in microbial structure between WKYs and SHRs. 16S rRNA gene sequencing was conducted on fecal samples from both groups. Significant differences were observed between the two groups (Figure 2A). Compared with WKYs, at the phylum level, the relative abundance of *Firmicutes* in SHRs increased (Figure 2B), while *Bacteroidetes*, *Actinobacteria*, and *Proteobacteria* decreased (*p* < 0.01). The ratio of *Firmicutes* to *Bacteroidetes* (F/B) was increased in SHRs (*p* < 0.0001) compared with WKYs (Figure 2C). The Microbial Dysbiosis Index (MDI), a reliable and reproducible index for assessing microbial dysbiosis (Kim et al., 2024), was positively correlated with the dysbiosis. The significantly increased MDI in SHRs indicated disordered gut microbiota (Figure 2D). The top 10 most abundant genera with differences between the two groups are shown in Figure 2E. *Bifidobacterium* and *Blautia* were significantly reduced in SHRs (*p* < 0.01), while *norank_f_norank_o_Clostridia vadinBB60 group*, *Lachnospiraceae_UCG-006*, *Colidextribacter*, and *DNF00809* were significantly more abundant in SHRs. Taken together, SHRs showed gut microbiota dysbiosis, which was represented by the reduction of potential beneficial bacteria.

**Figure 3:**
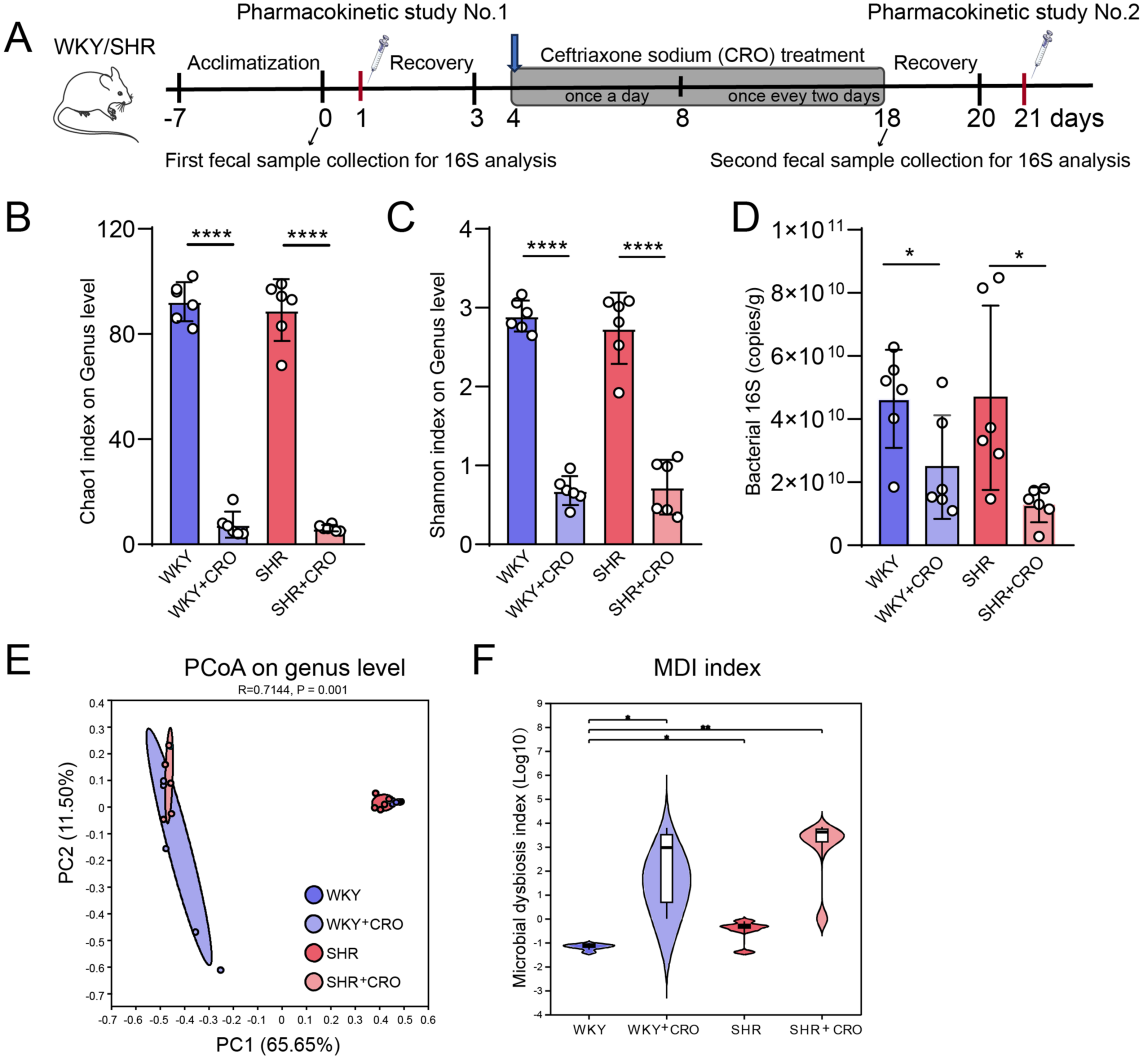
Gut microbiota analysis in WKYs and SHRs before and after treatment with ceftriaxone intervention. **(A)** Experimental design and sample collection in the study; **(B)** Chao1 and **(C)** Shannon index analysis chart of α diversity analysis; **(D)** Quantitation of bacterial load; **(E)** Principal Coordinates Analysis; and (D) MDI index of the four groups. Data are expressed as mean ± SD. ^*^p < 0.05, ^**^p < 0.01, ^***^p < 0.001, ^****^p < 0.001.

### 3.4 CRO-induced gut microbial dysbiosis further reduces the oral bioavailability of losartan in WKYs and SHRs

CRO was used to induce gut microbiota dysbiosis in rats to explore whether gut microbiota dysbiosis was related to the absorption and metabolism of losartan and its metabolites. During the CRO-induced period, we measured the body weight and blood pressure of rats, which showed no differences in body weight between the CRO-treated group and the untreated control group, both in WKYs and SHRs. While the SHR + CRO group showed a significant blood pressure increase on days 2 and 4 compared with the SHR group, there were no significant differences between the groups by the end of CRO administration (Supplementary Figure S5). Furthermore, to assess the effects of gut microbiota changes induced by CRO, we collected the second fecal samples from both WKYs and SHRs at the end of the period for 16S rRNA analysis (Figure 3A). According to the ASVs (Sobs) dilution curve, the gradual rarefaction curves of all samples were saturated as the number of sequences increased, indicating the data were reasonable and could be used for subsequent bioinformatics analysis (Supplementary Figure S6). Gut microbiota results showed that the treatment of CRO resulted in a significant reduction in α-diversity, represented by Chao1 (Figure 3B) and Shannon index (Figure 3C), and bacterial abundance (Figure 3D) in both WKYs and SHRs. Results of PCoA showed that the species composition of the CRO treated group was significantly different from that of the untreated group, both in WKYs and SHRs (Figure 3E). More importantly, a high MDI value indicated that CRO-induced intestinal flora dysbiosis was successfully established (Figure 3F).

**Figure 4:**
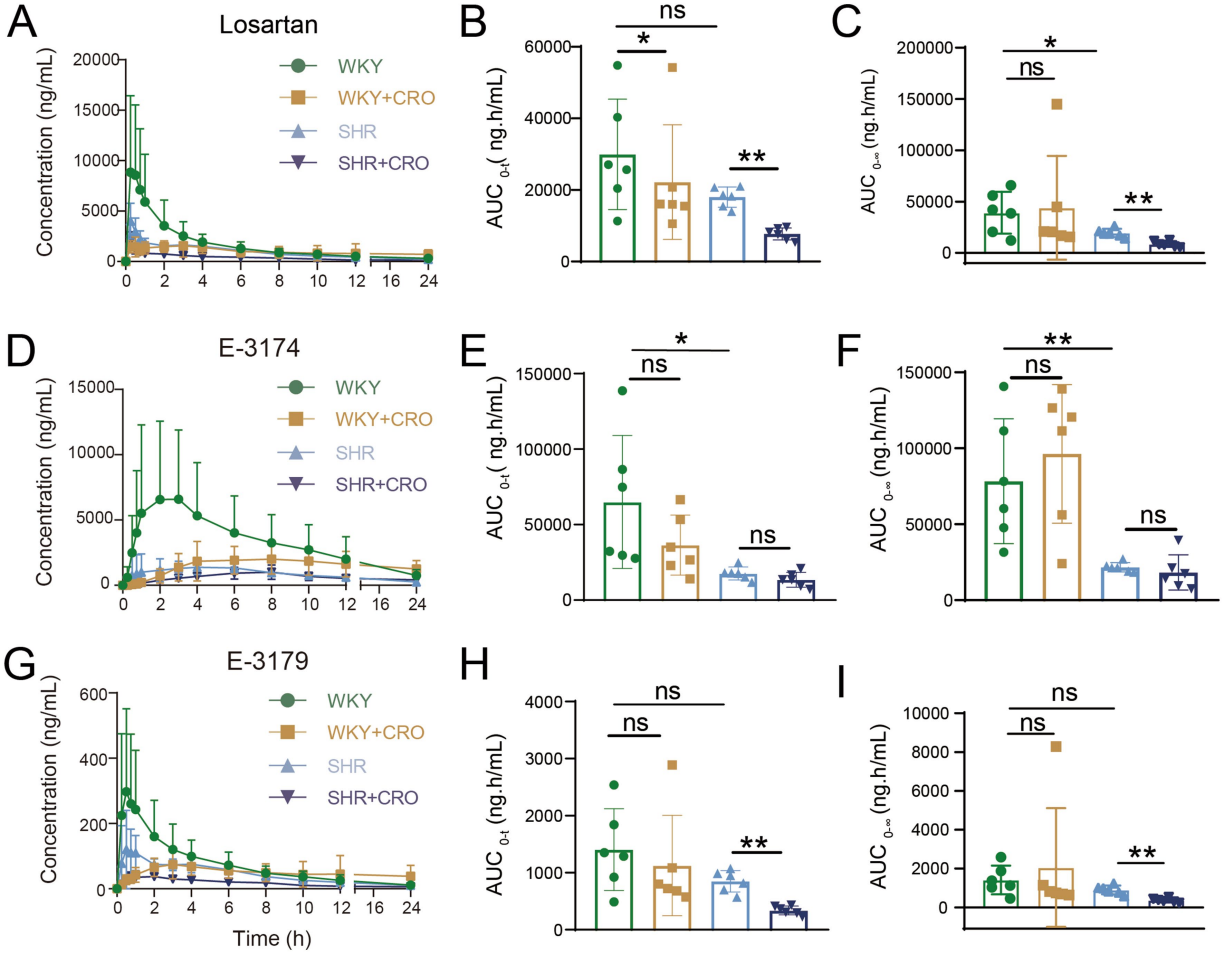
Pharmacokinetic parameters of losartan, E-3174, and E-3179 in WKYs and SHRs by LC–MS/MS. Plasma concentration-time curves within 24 h and a histogram for statistical analysis of the analyte pharmacokinetic parameters, AUC_0-t_ and AUC_0-∞_: **(A–C)** Losartan, **(D–F)** E-3174, and **(G–I)** E-3179. Data are expressed as mean ± SD. ^*^p < 0.05, ^**^p < 0.01, ns, no significance. Statistical analysis between the two groups was evaluated using an unpaired Student’s *t*-test with two-tailed distribution or a Mann–Whitney U test when data did not coincide with normal distribution, while a paired two-tailed Student’s *t*-test was applied to assess differences within the same group before and after the intervention with CRO administration.

Next, we conducted the second pharmacokinetic study on WKYs and SHRs (both treated with CRO). After oral administration of losartan potassium at the same doses, the same pharmacokinetic parameters were evaluated in these two CRO-treated groups. Compared with the pre-treatment groups, the blood concentration of losartan and its metabolites (E-3174 and E-3179) was changed by CRO treatment in both WKYs and SHRs (Figure 4), indicating that the bioavailability of losartan and its metabolites was modulated by CRO-induced intestinal microbial dysbiosis. Specifically, for losartan (Figures 4A–C), compared with the WKY group, the WKY + CRO group showed a 25.9% decrease in the AUC_0-t_ of losartan (29942.21 ± 15428.31 vs. 22185.17 ± 16026.77 ng·h/mL, *p* < 0.05). Similarly, compared with the SHR group, the SHR + CRO group exhibited a 57.20% reduction in the AUC_0-t_ of losartan (18002.60 ± 2806.38 vs. 7702.83 ± 1695.54 ng·h/mL, *p* < 0.01) (Table 1). However, CRO treatment did not significantly alter the AUC_0-t_ and AUC_0-∞_ values of E-3174 in either WKYs or SHRs, as shown in Figures 4D–F. In contrast, the SHR + CRO group showed significant reductions in the AUC_0-t_ and AUC_0-∞_ values of E-3179 compared to the SHR group (Figures 4G–I), with no significant differences observed between the WKY + CRO and WKY groups. Additionally, CRO-induced intestinal microbial dysbiosis affected other pharmacokinetic parameters. For instance, the WKY + CRO group showed significant reductions in the *C_max_* of losartan and E-3179 compared to the WKY group. The SHR + CRO group exhibited a significant decrease in E-3179 *C_max_* relative to the SHR group (Supplementary Figure S7). Notably, E-3174 had an extended MRT_0-t_ in both WKY + CRO and SHR + CRO groups compared to their untreated groups, and the MRT_0-t_ of losartan and E-3179 were notably prolonged in the WKY + CRO group compared to WKY (Supplementary Table S7). Collectively, these data demonstrated that CRO-induced gut microbiota dysbiosis not only reduced the oral bioavailability of losartan both in WKYs and SHRs, but also altered the pharmacokinetic profiles of losartan and its metabolites, including changes in *C_max_* and MRT_0-t_.

### 3.5 Association between gut microbiota changes induced by CRO treatment and pharmacokinetic parameters of losartan and E-3174

**Figure 5:**
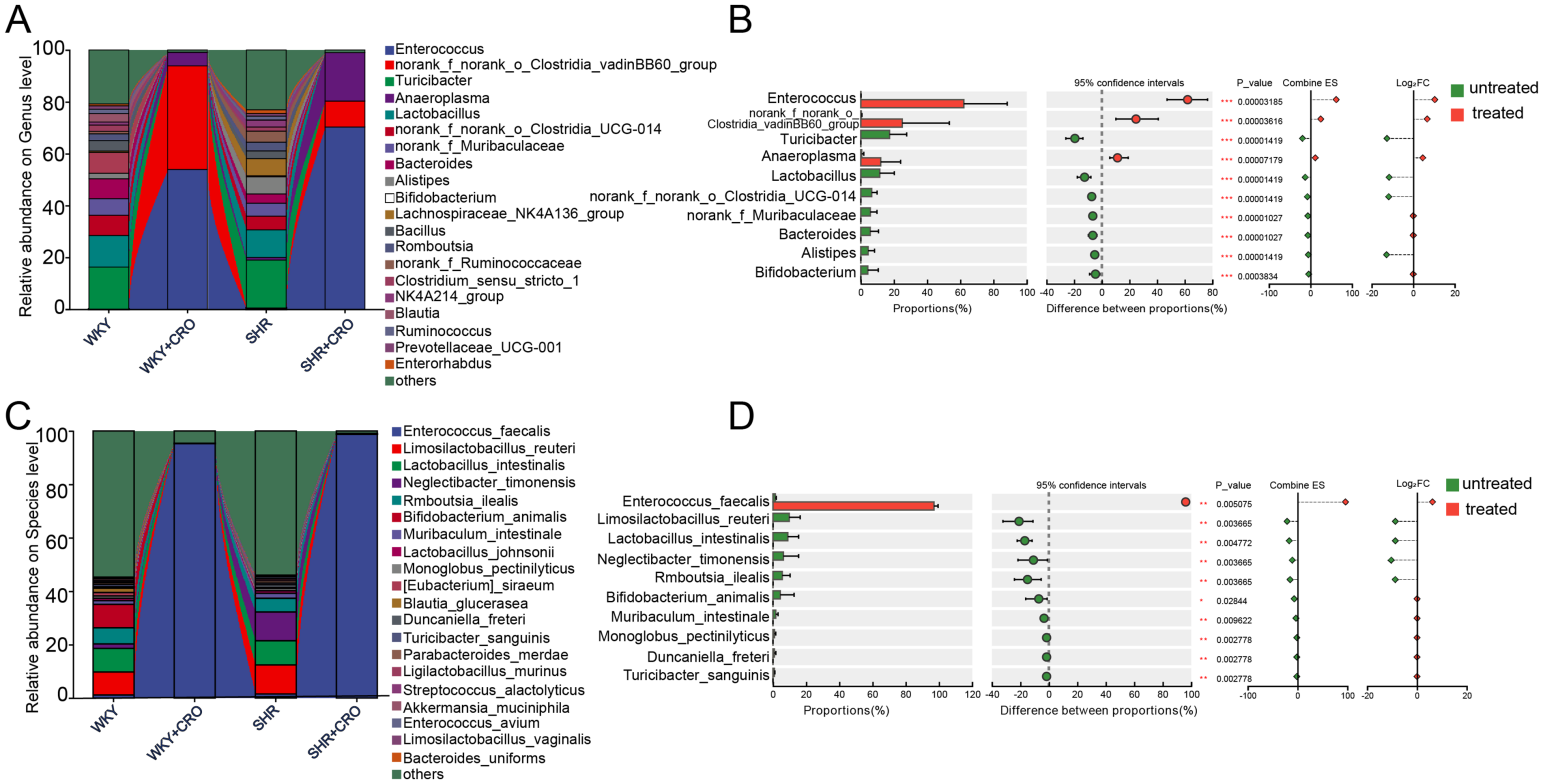
Fecal gut microbiota composition analysis altered by CRO. The top 20 bacteria by relative abundance and their differences between treated and untreated CRO groups at the **(A,B)** genus and **(C,D)** species levels. Data are expressed as mean ± SD. Wilcoxon signed-rank test bar plot on the genus or species level.

To evaluate the gut microbiota changes in rats induced by CRO, 16S rRNA sequencing and full-length 16S rRNA sequencing were performed on fecal samples. At the genus level, comparisons between different groups revealed that the relative abundance of *Enterococcus*, *norank_f_norank_o_Clostridia_vadinBB60_group*, and *Anaeroplasma* increased in both WKY + CRO and SHR + CRO groups compared to their untreated groups (Figure 5A). When rats were divided into untreated and CRO-treated groups, the top 10 most abundant genera with significant differences in content showed that *Enterococcus*, *norank_f_norank_o_Clostridia_vadinBB60_group*, and *Anaeroplasma* significantly increased, while the relative abundance of *Turicibacter*, *Lactobacillus*, *Bacteroides*, *Alistipes* and *Bifidobacterium* decreased (Figure 5B). At the species level, similar trends were observed. *Enterococcus faecalis* (*E. faecalis*) was the most abundant in both WKY + CRO and SHR + CRO groups (Figure 5C). In contrast, *Limosilactobacillus reuteri* (*L. reuteri*), *Lactobacillus intestinalis* (*L. intestinalis*)*, Bifidobacterium animalis* (*B. animalis*), and *Romboutsia ilealis* (*R. ilealis*) were more prevalent in the untreated group (Figure 5D). It was noted that the LDA value of *E. faecalis* was very high, reaching 5.68 (Supplementary Table S8); moreover, both *L. reuteri* and *E. faecalis* showed *p* < 0.05 in LEfSe analysis. LDA scores further confirmed the degree of enrichment of *Enterococcus faecalis* (Supplementary Figure S8).

**Figure 6:**
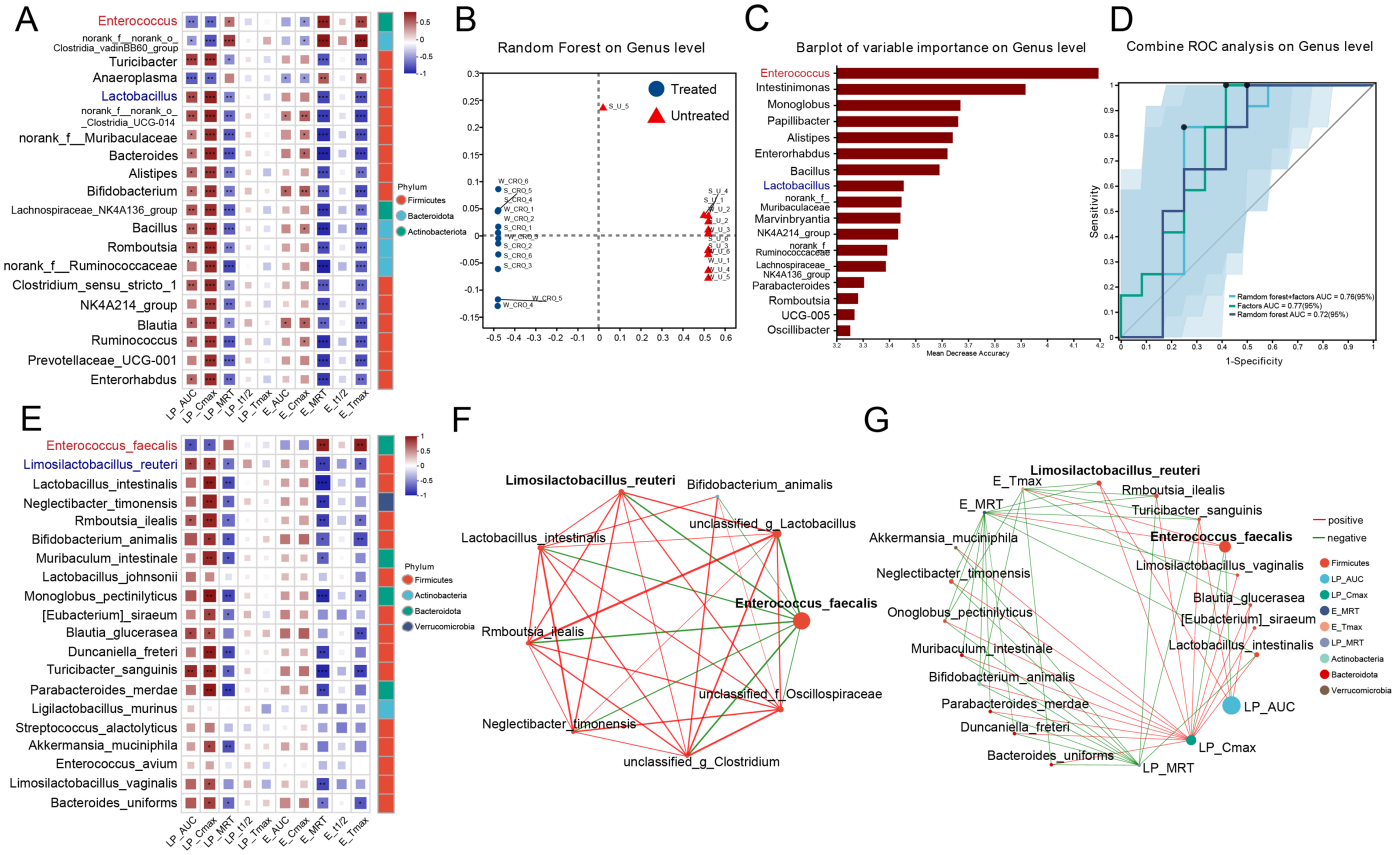
Correlations between gut microbiota and pharmacokinetic parameters. **(A)** Heatmap of correlation between the top 20 most abundant genera with content differences and pharmacokinetic parameters; **(B)** Random Forest; **(C)** Barplot of variable importance at the genus level and **(D)** ROC analysis; **(E)** Heatmap of correlation between the top 20 most abundant species with content differences and pharmacokinetic parameters; **(F)** univariate analysis and **(G)** Multivariate analysis at the species level. Spearman’s correlation analysis was used to evaluate the abundance of the most common bacteria at the genus level in the two groups. Only correlations with a *p*-value < 0.05 are shown. Color shading of nodes represents species abundance. Color gradation of lines represents the R value, with negative correlations shown in green and positive correlations shown in red.

Given the parallelism between pharmacokinetic characteristic changes and gut microbiota dysbiosis, we explored correlations between specific genera or species and pharmacokinetic parameters (AUC_0-t_, *C*_max_, *T*_max_, MRT_0-t_, and *T*_1/2_) of losartan and E-3174 in CRO-treated vs. untreated groups. Spearman’s correlation test was performed to assess the relationship between the top 20 most abundant genera and the pharmacokinetic parameters of losartan and E-3174 (Figure 6A). The results showed that after CRO treatment, *Enterococcus*, *norank_f_norank o_Clostridia_vadinBB60_group*, and *Anaeroplasma*, which significantly increased, negatively correlated with AUC_0-t_ and *C_max_* of losartan and E-3174 but positively with their MRT_0-t_ and *T*_max_. Conversely, *Turicibacter*, *Lactobacillus*, *Bacteroides*, *Alistipes*, and *Bifidobacterium*, which significantly decreased, positively correlated with AUC_0-t_ and *C_max_* of losartan and E-3174. Random forest algorithms were employed to distinguish between treated and untreated groups based on the relative abundance of selected bacterial genera (Figure 6B). Key discriminative genera included *Enterococcus*, *Intestinimonas*, *Alistipes*, *Enterorhabdus*, *Bacillus*, and *Lactobacillus* (Figure 6C). The top 20 genera showed a strong ability to identify CRO induced changes with AUC values of 0.72, 0.77, and 0.76 when using distinguishing microbiota, pharmacokinetic parameters, or both (Figure 6D). Furthermore, the lower limit of the 95% CI was above 0.5, indicating that the ROC has high reliability in pharmacokinetic parameters and Random Forest combined with pharmacokinetic parameters. 16S rRNA gene full-length sequencing further substantiated that *E. faecalis* negatively correlated with losartan’s AUC_0-t_ and *C_max_*. In contrast, *L. reuteri*, *Rmboutsia_ilealis* (*R. ilealis*), *Blautia_glucerasea* (*B. glucerasea*) and *Turicibacter_sanguins* (*T. sanguins*) positively correlated with these parameters (Figure 6E). The univariate correlation network diagram showed that *E. faecalis* negatively correlated with strains positively related with AUC_0-t_ of losartan, including those mentioned above (Figure 6F). The two-factor correlation network diagram further indicated that higher *E. faecalis* abundance negatively correlated with the AUC_0-t_ and *C_max_* of losartan, and positively correlated with the MRT_0-t_ and *T*_max_ of E-3174 (Figure 6G), underscoring its role in CRO-induced dysbiosis and subsequent pharmacokinetic perturbations.

### 3.6 *Enterococcus faecalis* significantly degrades losartan and converts it into E-3179 *in vitro*

**Figure 7:**
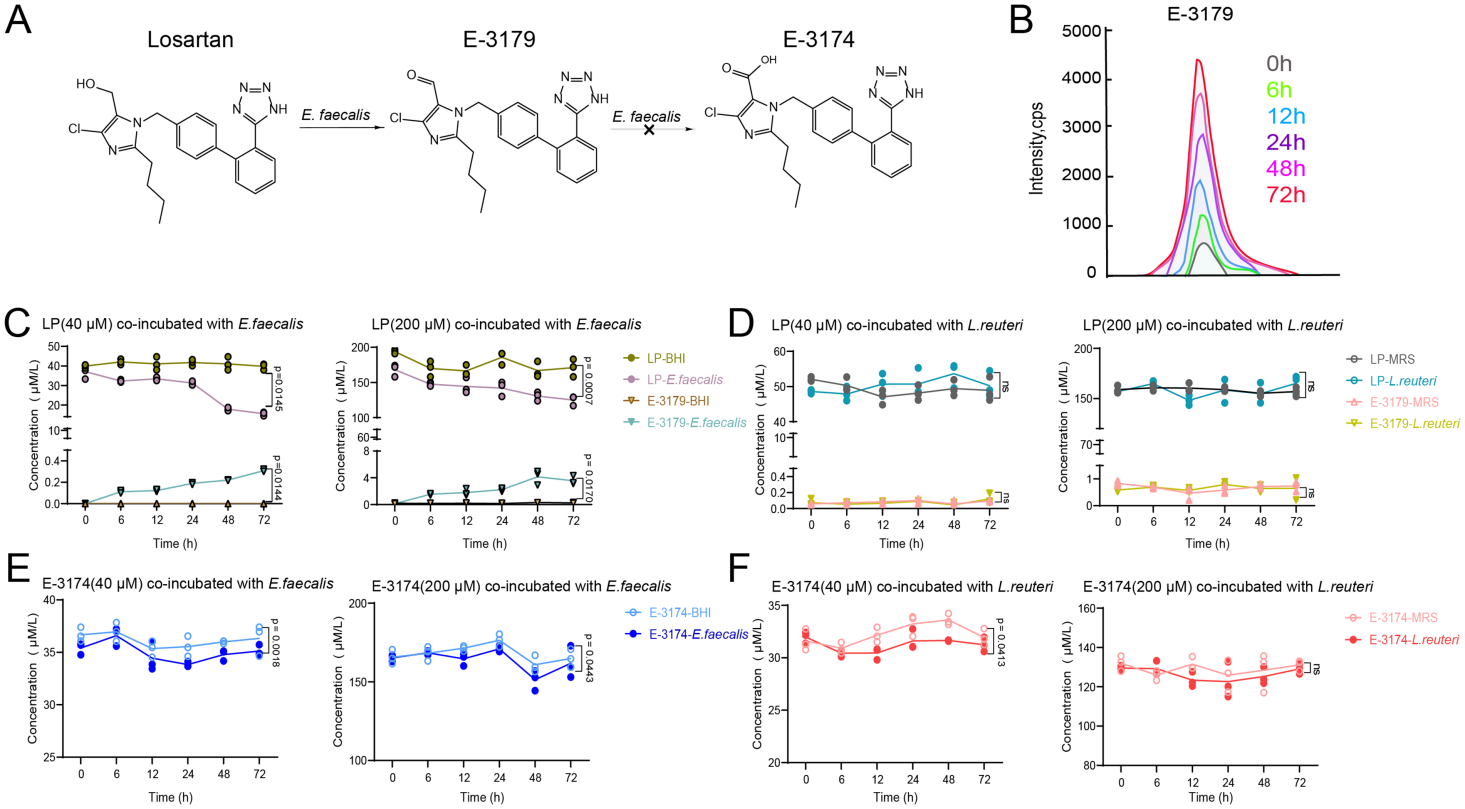
Degradation and transformation of losartan and E-3174 by *E. faecalis* and *L. reuteri*. **(A)** *In vitro* degradation and conversion of losartan by *E. faecalis*. **(B)** Intensity of E-3179 when losartan was co-incubated with *E. faecalis*. Remaining content of losartan (40 μM and 200 μM) when co-incubated with **(C)** *E. faecalis* and **(D)** *L. reuteri*. Remaining content of E-3174 (40 μM and 200 μM) when co-incubated with **(E)** *E. faecalis* and **(F)** *L. reuteri*. Data are expressed as mean ± SD.

To assess how *E. faecalis* affected losartan bioavailability, we conducted *in vitro* tests with losartan and E-3174, using *L. reuteri* as a control strain. Each bacterial strain was incubated separately with these two drugs under their growth conditions. Concurrently, drug-free and bacteria-free controls were included. Supernatants were collected at various time points: 0, 6, 12, 24, 48, and 72 h, and analyzed via LC–MS/MS. Results indicated that *E. faecalis* degraded losartan into E-3179 but not further into E-3174 (Figure 7A). This was confirmed by increased E-3179 levels over time (Figure 7B) and with higher losartan concentrations (Figure 7C). In contrast, *L. reuteri* exhibited no significant degradation capability for losartan (Figure 7D). Besides, *E. faecalis* exhibited significant degradation of E-3174 at both 40 and 200 μM (Figure 7E), while *L. reuteri* degraded E-3174 at 40 μM (Figure 7F). Additionally, the tested concentrations of losartan and E-3174 did not inhibit the growth of these two bacterial strains (Supplementary Figure S9). Collectively, these findings showed that *E. faecalis* directly degrades losartan and E-3174 *in vitro*, a process that might be the main cause of reduced oral bioavailability in rats following CRO treatment, driven by microbial metabolic interference.

## 4 Discussion

The pharmacokinetics of losartan potassium exhibit significant variability among individuals (Sica et al., 2005), which was usually attributed to genetic polymorphism of the CYP2C9 enzyme (Park et al., 2021) and structural variability of AGTR1 (Zeng et al., 2023). This pharmacokinetic variability may, to some extent, affect blood pressure control in clinical treatment (Xiong et al., 2021). Recent evidence has highlighted the role of gut microbiota in hypertension, with dysbiosis associated with the development of hypertension (Richards et al., 2017; Qi et al., 2017). Despite the need for more aggressive interventions for hypertension, response rates to monotherapy with any antihypertensive drug remain around 50% (Materson et al., 1993). Furthermore, approximately 15% of this rapidly growing population is resistant to all interventions (Benjamin et al., 2017), emphasizing the need for diverse and innovative strategies for hypertension treatment. In this context, the interaction between microbiota and drugs has been underestimated (Curini and Amedei, 2021). While the vast metabolic capacity of the microbiome to metabolize a diverse array of drugs is clear, the extent to which changes in the microbiome, especially in terms of dysbiosis, can result in clinical changes in systemic drug levels remains unexplored (Cussotto et al., 2021).

In the present study, we explored the effect of gut microbiota dysbiosis, from endogenous to CRO-induced gut microbiota dysbiosis, on the metabolism and pharmacokinetics of losartan potassium. Our results revealed that losartan oral bioavailability was significantly decreased in gut microbiota dysbiosis rats, when SHRs were compared to WKYs, and this was further validated in a rat CRO-induced gut microbiota dysbiosis model. The reduction in losartan oral bioavailability resulting from CRO-induced dysbiosis was associated, to some extent, with the direct degradation and conversion of losartan to E-3179 by *E. faecalis*.

Abundant evidence has shown that gut microbiota can metabolize drugs (Martinelli and Thiele, 2024). To explore the role of microbiota in losartan pharmacokinetic variability, we first co-incubated losartan and E-3174 with fecal samples from WKY and SHR rats. Results indicated that both compounds were degraded by the microbiota, aligning with prior findings of losartan degradation by human intestinal flora (Zimmermann et al., 2019). Notably, degradation was more pronounced with SHR microbiota in our study. To assess whether WKYs and SHRs differ in the *in vivo* metabolism of losartan potassium, we performed an in vivo experiment. After oral administration of losartan potassium, metabolites in plasma, feces, and urine were screened using UHPLC-Q-TOF-MS. The analysis identified not only known losartan metabolites (E-3179 and E-3174) but also a compound with an exact mass of 439.16, which existed as four isomers in both plasma and feces. Based on mass spectral behavior, we speculated that two of these isomers were metabolites of losartan with a butyl side chain (Stearns et al., 1992; Chen et al., 1993), while the other two isomers were newly identified. Furthermore, our comparison with previous work demonstrated that these latter isomers were not oxidative degradation products of losartan (Xia et al., 2024). Notably, the four isomers were more abundant in feces than in plasma, indicating that they were likely derived from losartan via gut microbiota. Furthermore, losartan and its metabolites were more abundant in the feces and plasma of WKYs than in SHRs, suggesting distinct metabolic capacities between the two groups.

Subsequently, we conducted pharmacokinetic analyses to evaluate the differential absorption and metabolism of losartan and its metabolites in different rats. The present study revealed a significant decrease in the AUC_0-∞_ of losartan and its active metabolite E-3174 in SHRs compared to WKYs, indicating reduced oral bioavailability of these compounds. Further 16S rRNA sequencing revealed gut microbial dysbiosis in SHRs, with higher F/B ratio and MDI than WKYs—which aligned with previous findings (Yang et al., 2015). These results together implied that gut microbial dysbiosis leads to differences in the absorption and metabolic capacity for losartan and its metabolites between WKYs and SHRs. Moreover, given the metabolic role of the CYP2C9 enzyme in losartan, we measured CYP2C9 enzyme activity in rat liver using a commercial kit (Rat cytochrome P450-2C9 enzyme YG10258, MlBio). Although there was no significant difference in CYP2C9 enzyme activity between the WKY and SHRs (Supplementary Figure S10), the current results do not rule out the potential influence of this enzyme. Further investigation of CYP2C9 expression at the mRNA and protein levels is required. Next, we performed Spearman’s correlation analysis between the altered gut microbiota and the pharmacokinetic parameters of losartan and E-3174 to explore whether the decreased bioavailability might be attributed to the degradation of losartan by specific gut bacteria—given the observed gut microbial dysbiosis in SHRs and the known metabolic capacity of gut microbiota for xenobiotics. However, no specific strains that were significantly enriched in SHRs and positively correlated with reduced losartan absorption were detected. Thus, we induced gut microbiota dysbiosis using ceftriaxone sodium on the same batch of rats to further investigate its role in the pharmacokinetics of losartan and its metabolites.

Ceftriaxone sodium is commonly used as an antibiotic to treat bacterial infections (Francioli, 1993). Administered intravenously or intramuscularly, it can reach high concentrations in the gastrointestinal tract via biliary excretion. This process disrupts microbial colonization resistance (Kokai-Kun et al., 2020), alters gut microbiota composition, and thereby induces intestinal dysbiosis (Almugadam et al., 2021; Wang et al., 2025). Here, we used it as a dysbiosis inducer. 16S rRNA sequencing results showed that CRO administration significantly decreased fecal microbial diversity and bacterial load, inducing dysbiosis in both WKY and SHR rats. Principal component analysis of beta diversity further indicated marked differences in gut microbial communities between CRO-treated and untreated rats. Importantly, pharmacokinetic results revealed a significant reduction in the oral bioavailability of losartan and E-3179 in the SHR + CRO group compared to SHRs, while the MRT_0-t_ of losartan, E-3174, and E-3179 was significantly prolonged in the WKY + CRO group compared to WKYs. These findings demonstrated that gut microbial dysbiosis reduced the absorption of losartan and its metabolites and altered their metabolic kinetics.

To explore the relationship between specific bacteria altered by CRO and pharmacokinetics parameters, we performed 16S rRNA and 16S full-length sequencing on fecal samples. At the genus level, *Enterococcus* was the most significantly enriched based on the variable importance genus in CRO treated rats; at the species level, this enrichment was attributed to *Enterococcus faecalis*. This finding aligned with a previous study showing that CRO treatment promotes *E. faecalis* colonization in the mouse gut (Chakraborty et al., 2018). Conversely, we observed a significant reduction in beneficial bacteria, such as *L. reuteri* and *B. animalis*. We further performed Spearman correlation analysis to link microbial changes with pharmacokinetic parameters. Results showed a significant negative correlation between *E. faecalis* abundance and losartan AUC_0-t_, while *L. reuteri*, *R. ilealis*, *B. glucerasea*, and *T. sanguinis* abundance was positively correlated with these parameters. To understand the potential mechanism of these changed intestinal flora, PICRUSt2 combined with KEGG analysis showed that the bacterial functions before and after CRO treatment differ in the metabolic pathways of Microbial metabolism in diverse environments, Biosynthesis of amino acids, Carbon metabolism, Ribosome, and ABC transporters (Supplementary Figure S11). Previously, a study reported that the reduced efficacy of the antihypertension drug captopril in rats was attributed to direct metabolism of the drug by a specific intestinal bacterium (Yang et al., 2022). To explore the biotransformation of losartan by microbiota, we conducted the *in vitro* experiments, which found that *E. faecalis* significantly degraded losartan and E-3174, with partial conversion of losartan to E-3179; in contrast, as a control strain, *L. reuteri* showed no significant metabolic activity toward these compounds. Notably, *Enterococcus* had been reported to be dominant in hypertensive patients with drug resistance (Guo et al., 2024), and Sprague–Dawley rats receiving live *E. faecalis* but not dead bacteria exhibited higher blood pressure (Zhu et al., 2021). Conversely, *L. reuteri* supplementation had been shown to improve blood pressure in SHRs (Xiong et al., 2024). Combined with these findings, it was concluded that gut dysbiosis induced by CRO reduced the oral bioavailability of losartan. This effect may be mediated by the degradation of losartan by *E. faecalis*, with partial conversion to the inactive metabolite E-3179. Furthermore, it should be noted that gut microbiota imbalance in hypertensive patients is complex; it can be caused by many factors, such as diets, comorbidities (e.g., diabetes), and unhealthy lifestyles. It manifests as microbial community dysregulation, with altered pathogenic-probiotic interactions. From this perspective, the gut microbiota imbalance was broad and requires contextual specification. Overall, these results provided evidence that gut microbial dysbiosis contributes to inter-individual variability in losartan’s efficacy in clinical research.

A limitation of this study is that the role of hepatic CYP enzyme activity in reducing losartan’s oral bioavailability remains unclear. Additionally, whether differences in intestinal metabolic enzymes and drug transporters exist between animals with various gut microbiota dysbiosis and their controls, and whether such differences contribute to the decreased bioavailability, is also uncertain. Notably, while the ceftriaxone-induced gut dysbiosis in this study directly increased *E. faecalis*, reflecting a direct effect of dysbiosis, indirect effects, such as on drug-metabolizing enzymes and transporters, cannot be excluded and require further investigation. Furthermore, although the significant role of *E. faecalis* had been established, further validation involving animal models targeting *E. faecalis* colonization was required to confirm that its direct effect in reducing losartan’s oral bioavailability. Additionally, mechanistic studies on *E. faecalis* and its relevant enzymes, as well as functional validation, are needed. Furthermore, metagenomic shotgun sequencing and multi-omics experiments, combined with advanced pipelines, could help to deeply explore the role of this species. More importantly, given the disparities between experimental models and humans, it is necessary to validate whether the results of this study can be extrapolated to humans, accounting for the specific contexts of gut microbiota dysbiosis. Notably, a drug’s effective concentration is fundamental to its pharmacodynamic effects. Integrating reduced bioavailability and altered gut microbiota will better explain individual differences in clinical drug responses and hypertension treatment efficacy.

## 5 Conclusion

By combining *in vitro* and *in vivo* experiments, our study explored the relationship between the antihypertensive drug losartan and gut microbiota dysbiosis in rats. This study showed that rats with gut microbiota dysbiosis exhibited altered metabolism and pharmacokinetics of losartan. Specifically, at the endogenous dysbiosis level, SHRs showed significant reduction in the bioavailability of losartan and its active metabolite E-3174 compared to WKYs. Furthermore, when dysbiosis was induced by CRO, losartan’s oral bioavailability was further decreased in both WKYs and SHRs. Mechanistically, CRO-induced changes in the pharmacokinetics of losartan might be associated with the degradation and conversion of losartan by *E. faecalis*. Our study highlighted that gut microbiota dysbiosis significantly impacts the metabolism and pharmacokinetics of losartan and its metabolites. These findings suggest a microbial-oriented approach to explain the inter-individual variability in clinical response to antihypertensive drugs. Future research should focus on translating these animal experimental results into clinical practice and exploring strategies to reshape the gut microbiota to enhance drug bioavailability.

## Data Availability

The datasets presented in this study can be found in online repositories. The names of the repository/repositories and accession number(s) can be found in the article/Supplementary material.
