# Supplementary material for: Effects of gut microbiota dysbiosis on the metabolism and pharmacokinetics of losartan in rats: from endogenous to ceftriaxone-induced dysbiosis
Source: Front Microbiol. 2025 Dec 2;16:1693247. doi: 10.3389/fmicb.2025.1693247 (PMC12705613; doi:10.3389/fmicb.2025.1693247)
Supplement: Supplementary file 1 [file Table_1.DOCX]

***Supplementary Material***

**Effects of gut microbiota dysbiosis on the metabolism and pharmacokinetics of losartan in rats: from endogenous to ceftriaxone-induced dysbiosis**

1. **Supplementary Method**
   1. **Sample Preparation of Supernatants from Microbiota-Drug Co-Incubation for Qualitative and Quantitative Analysis**

The sample processing procedures were tailored to align with the experimental objectives. For qualitative metabolite profiling, supernatants from the co-incubation of bacteria and drugs were collected at designated time points, filtered using a 0.22 μm membrane, lyophilized, and reconstituted in 200 μL of methanol. After vortexing and centrifuging for 10 minutes at 4°C and 12,000 rpm, the supernatant was transferred to an LC vial, and 5 μL aliquot was injected into the UHPLC-Q-TOF-MS/MS. For quantitative analysis, 100 μL aliquots were taken from the co-incubation supernatant and spiked with 1,000 μL of methanol containing 500 ng/mL of irbesartan. Following one minute of vortexing and centrifugation at 12,000 rpm for 10 minutes, the resulting supernatant was transferred to an LC vial, and a 5μL aliquot was injected into the UHPLC-TQ-MS/MS.

- 1. **Sample Preparation of In Vivo Metabolites for Qualitative Analysis**

The biological samples were processed as follows: For plasma, a total of 400 μL of pooled plasma was mixed by combining 50 μL aliquots from each time point. A 100 μL aliquot was mixed with 300 μL of methanol, vortexed for one minute, and centrifuged at 5,000 rpm for 10 minutes at 4°C [1]. In the case of urine, 1 mL was centrifuged at 4,000 rpm for 20 minutes at 4°C, and the supernatant was mixed with 2 mL of methanol, vortexed for one minute, and centrifuged at 12,000 rpm for 10 minutes at 4°C [2]. For fecal samples, 1.0 g of homogenized feces was extracted with 4 mL of methanol, sonicated in an ice-water bath for 20 minutes, and then centrifuged at 12,000 rpm for 10 minutes at 4°C [3]. Supernatants from the plasma, urine, and fecal extracts were evaporated under nitrogen gas, redissolved in methanol, and transferred to liquid chromatography vials. Samples were analyzed using UHPLC-Q-TOF-MS/MS for metabolite profiling.

- 1. **UHPLC-Q-TOF-MS/MS Analysis**

UHPLC-Q-TOF-MS/MS was performed on a SCIEX Exion LC™ AD Series LC-ESI-Q-TOF-MS (X500R QTOF, AB SCIEX, Foster City, CA) equipped with a Turbo ion source and ESI probe. Sciex OS 2.1 software was applied for data acquisition and evaluation. MS detection was performed using IDA methods with ESI ionization in positive mode. Tuning solutions for both ESI positive and negative modes were used to automatically calibrate the mass spectrometer. The generic source parameters are shown in Supplementary Table 1. The LC separation was achieved through a gradient elution with 0.5% acetic acid in water (solvent A) and 0.5% acetic acid in acetonitrile (solvent B). The flow rate was set at 0.2 mL min^-1^, and the gradient elution was performed as follows: 0 min - 22% B, 1 min - 22% B, 3 min - 24% B, 6 min - 26% B, 10 min - 30% B, 10.5 min - 30% B, 12 min - 35% B, 14 min - 40% B, 15 min - 40% B, 19 min - 85% B, 22 min - 85% B, 22.1 min - 22% B, 25 min - 22% B. The sample injection volume was 5 μL, and the column temperature was maintained at 40°C.

- 1. **UHPLC-TQ-MS/MS Analysis**

### 1.4.1 Detection Method

For the determination of drug remaining contents in the supernatant, the gradient elution was as follows: 0.1–0.5 min at 45% B; 0.5–1.0 min at 45–65% B; 1.5–4.0 min at 65–85% B; 4.0–4.5 min at 85% B; 4.5–4.6 min at 85%–45% B; 4.6–5.0 min at 45% B. For pharmacokinetics, the gradient elution was performed as follows: 0–0.5 min at 30% B; 0.5–1.5 min at 30–45% B; 1.5–5.0 min at 45–60% B; 5.0–7.0 min at 60–80% B; 7.0–8.0 min at 80% B; 8.1–10.0 min at 30% B. The injection volume was 5 μ L. The multiple reaction monitoring (MRM) mode was used to detect analytes and IS in the positive ion mode. The mass spectrometry parameters were set as follows: ion spray voltage was 5500 V, turbo spray temperature (TEM) of 550℃; nebulizer gas (gas 1) of 50 psi; heater gas (gas 2) of 50 psi and curtain gas of 40 psi. Nitrogen was kept as the nebulizer and auxiliary gas. The detailed MRM channel, collision energy (CE) and declustering potential (DP) were given as follows: losartan, 423.1→207.2, CE: 30 V, DP 92 V; E-3174, 437.2→207.1, CE: 32 V, DP: 154 V; E-3179, 421.1→207.2, CE: 33 V, DP: 63 V; and IS 429.2→207.1, CE: 36 V, DP: 128 V. The dwell time of each MRM channel was 100 ms.

### 1.4.2 Preparation of Calibration Standards and Quality Control Samples

The standards of losartan, E-3174, E-3179, and IS were accurately weighed and dissolved in methanol to obtain their standard stock solutions with a final concentration of 2.0, 3.0, 1.0 and 5.0 mg/mL, respectively. The standard stock solutions of losartan, E-3174, and E-3179 were further diluted with methanol to obtain the mixture of working solutions at a series of concentration levels. The IS working solution final concentration is 500 ng/mL. The calibration standards samples were prepared by spiking 10 μL of the corresponding mixture working solutions with 100 μL of blank plasma to obtain concentrations levels of 0.1, 0.5, 10, 100, 1000, 4000 and 10000 ng/mL for losartan; 5, 25, 50, 250, 500, 2000 and 5000 ng/mL for E-3174; 0.2, 1, 10, 100, 200, 800 and 2000 ng/mL for E-3179 finally. Quality control (QC) samples were prepared in blank rat plasma with losartan, E-3174 and E-3179 respectively, at lower limit of quantitation (LLOQ, 0.1, 5, 0.2 ng/mL), low QC (LQC, 0.2, 10, 0.4 ng/mL), middle QC (MQC, 2000, 1000, 400 ng/mL), and high QC (HQC, 8000, 4000, 1600 ng/mL). All the standard stock solutions, working solutions, calibration standards, and QC samples were stored at -20 ℃ until analysis.

### 1.4.3 Method Validation

#### 1.4.3.1 Selectivity and Sensitivity

The selectivity of the method was evaluated by comparing the six blank plasma samples with the LLOQ samples of analytes and pre-extraction spiked plasma samples (500 ng/mL) of IS. Peak areas of the endogenous interference in the blank samples should be lower than 20% of the peak areas for the analytes in LLOQ samples and be lower than 5% of the peak area for IS in pre-extraction spiked plasma samples. The sensitivity of this method required that the LLOQ of losartan, E-3174, and E-3179 could cover the drug concentration at the pharmacokinetic time points. Meanwhile, the signal-to-noise ratio (SNR) of analyte peaks should be close to 10 in the LLOQ samples.

#### 1.4.3.2 Carryover

The carryover was estimated by injecting a blank plasma sample after injection of the upper limit of quantification (ULOQ) samples. The residue of analytes and IS detected in the blank plasma sample should not exceed 20% of LLOQ and not exceed 5% of IS.

#### 1.4.3.3 Linearity, Precision and Accuracy

The linearity of losartan, E-3174, and E-3179 was evaluated using their calibration curves from three independent batches according to their calibration standards in the range of 0.1–10000 ng/mL. The calibration curves were obtained by plotting the peak area ratio (y) of analytes to IS against their nominal concentration (x) using a weighting factor (1/x^2^) in linear regression. The correlation coefficient (R^2^) of each calibration curve should be guaranteed to be higher than 0.99. The intra-day and inter-day precision and accuracy were performed by analyzing six replicates of LLOQ, LQC, MQC, and HQC samples in three continuous batches over at least 2 days. The precision of determination was expressed as the relative standard deviation (RSD), and the accuracy was described as the relative error (RE), which was calculated as (observed concentration - nominal concentration) / theoretical concentration×100%. The RSD and RE should be adjusted within ± 15% for QC samples and within ± 20% for LLOQ samples.

#### 1.4.3.4 Extraction Recovery and Matrix Effect

The matrix effect was assessed by evaluating six replicates of LQC, MQC, and HQC using six different sources of blank rat plasma to identify the interference of endogenous components of rat plasma on the ionization of losartan, E-3174, E-3179, and the IS. The matrix effect was determined by comparing the mean peak areas of analytes spiked in the extracted blank plasma (set 1) with those of a neat solution of the compound in methanol (set 2), at equivalent concentrations. The extraction recovery was evaluated to determine the efficiency and reproducibility of the sample extraction process. The peak areas of the extracted samples (set 3) were compared with those of the set 1 sample.

#### 1.4.3.5 Stability

To examine the stability of losartan, E-3174, and E-3179 in biological matrix under different conditions, stability experiments including the autosampler stability, thaw stability (three cycles), short-term stability, and long-term stability were investigated at LQC and HQC samples (six replicates at each level). Analysis of QC samples was performed according to a standard curve, which was obtained from freshly prepared calibration standard samples. The autosampler stability was determined by placing samples in the autosampler rack at 8 ℃ for 24 h. The freeze-thaw stability was evaluated after three complete freeze-thaw cycles (-80 ℃ to 25 ℃) on consecutive days. The short-term stability was determined after samples were exposed to room temperature for 6 h. The long-term stability was assessed after storing the samples at -80 ℃ for 30 days. The analytes were considered stable if the deviation between the mean measured concentration and the nominal concentration was within ± 15.0%.

#### 1.4.3.6 Dilution Integrity

Dilution integrity experiments were conducted at 10, 1000, and 100,000 times the ULOQ concentration. Six replicate pre-extraction spiked plasma samples at different concentrations were diluted with 10% blank plasma using different dilution factors (Supplementary Table 5). Their concentrations were determined using freshly prepared calibration curves for losartan, E-3174, and E-3179. For these diluted samples, the RSD should not exceed 15% and the RE should be within ± 15%.

1. **Supplementary Results**
   1. **Optimization of UHPLC-TQ-MS/MS Method**

Samples of plasma preparation methods were optimized, including extraction by ethyl acetate-hexane–ethyl ether ^[4]^, HCl processing followed by diethyl ether ^[5]^, combined with protein precipitation using acetonitrile ^[6]^ or methanol ^[7]^. Finally, protein precipitation using methanol was selected due to higher analyte abundance and ease of processing. The mobile phase consisted of 0.1% formic acid in water and acetonitrile, with a ten-minute gradient elution at 40°C and a flow rate of 0.3 mL/min. MS parameters were optimized using reference solutions, and the optimal settings were determined using an AB SCIEX instrument optimization (B.04.01).

- 1. **UHPLC-TQ-MS/MS Method Validation**

The developed method was validated, including selectivity, linearity, carryover, accuracy and precision, sensitivity, matrix effects, extraction recovery, dilution effect, and stability, following the US FDA guidelines. Representative MRM chromatograms of analytes and IS were shown in Supplementary Figure 4; no endogenous interference peaks were detected at the retention times of all analytes or the IS, indicating the good selectivity and high sensitivity of the method. All analytes exhibited good linear regression with correlation coefficients no less than 0.9909 over a range of 0.1 to 10,000 ng/mL (Supplementary Table 3). The signal-to-noise ratios (SNR) at the lower limit of quantification (LLOQ) were 21.01, 17.70, and 12.14 for losartan, E-3174, and E-3179, respectively. The carryover for losartan, E-3174, E-3179, and IS was less than 4.24%, 0.00%, 3.57%, and 0.01%, respectively, which ensured no interference between the consecutive samples in batch operation. Intra-day and inter-day precision and accuracy were within acceptable limits, indicating the reliability and reproducibility of the method. Extraction recovery ranged from 95.52% to 108.68%, and matrix effects with RSD ranged from 1.06% to 7.46% (Supplementary Table 4). Stability showed that all analytes were stable under routine conditions, with deviation not exceeding 12.98% (Supplementary Table 5). The dilution integrity was validated, with RSD and RE values less than 5.28% and 11.54%, respectively (Supplementary Table 6). All the method validation results indicated that the UHPLC-TQ-MS/MS method was dependable for determining the concentrations of losartan, E-3174, and E-3179 in rat plasma.

1. **Supplementary Figures and Tables**
   1. **Supplementary Figures**


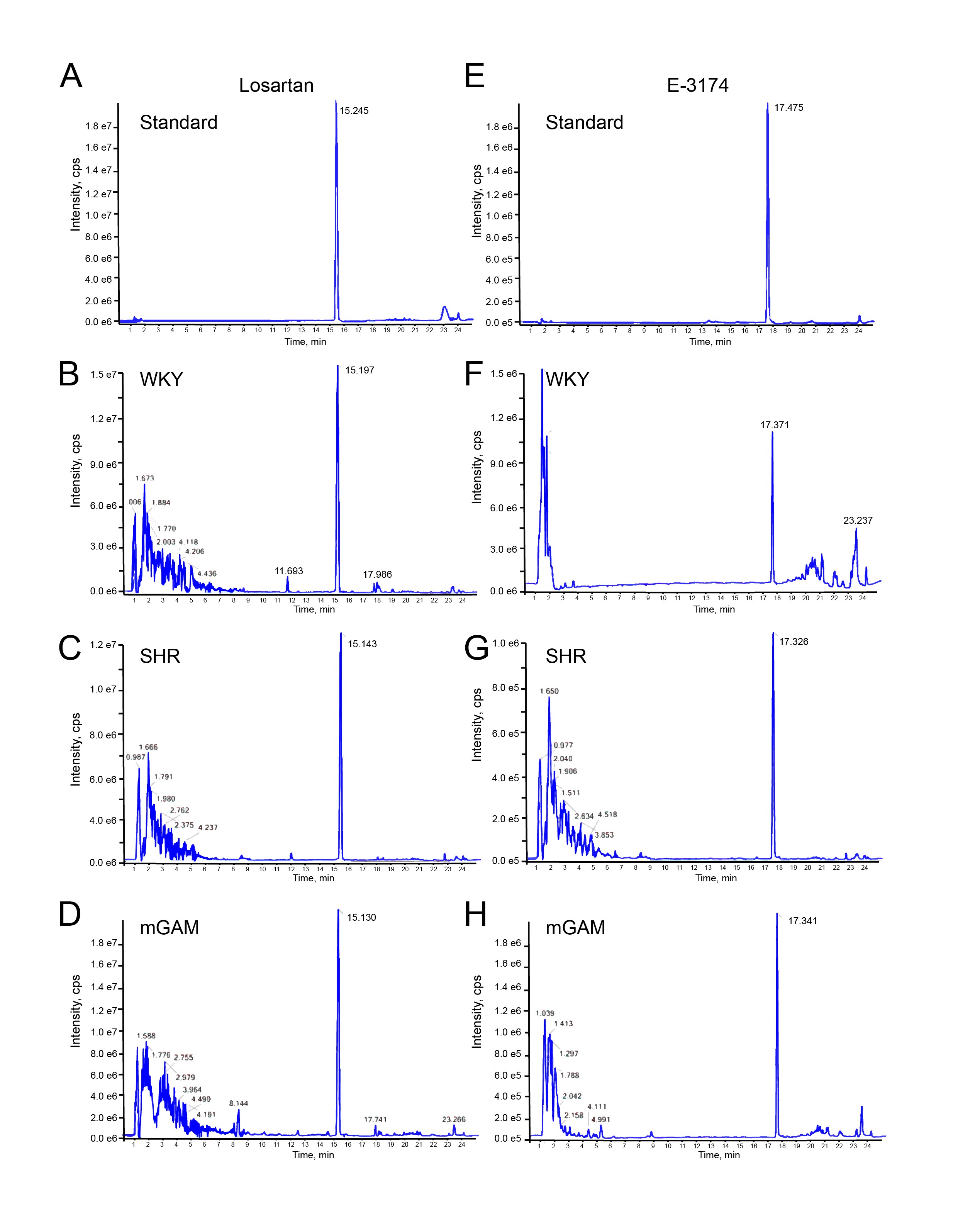


Supplementary Figure 1. Total ion chromatograms of losartan (A-D) and E-3174 (E-H) after co-incubation with fecal bacteria of WKY and SHR rats.


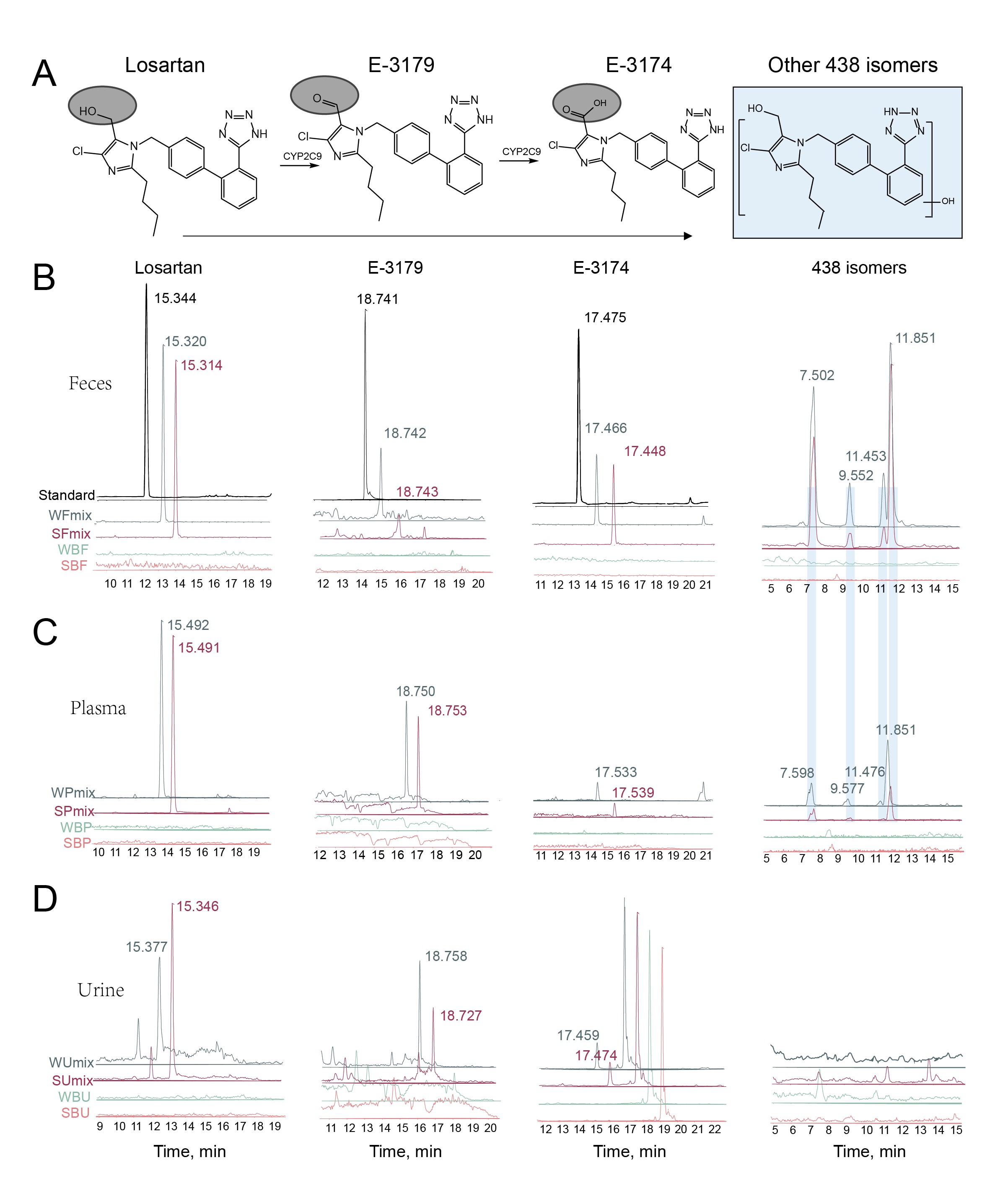


Supplementary Figure 2. Identification and characterization of metabolites in vivo in WKYs and SHRs. (A) Conversion process of losartan; (B) Metabolites in feces; (C) Metabolites in plasma; (D) Metabolites in urine.


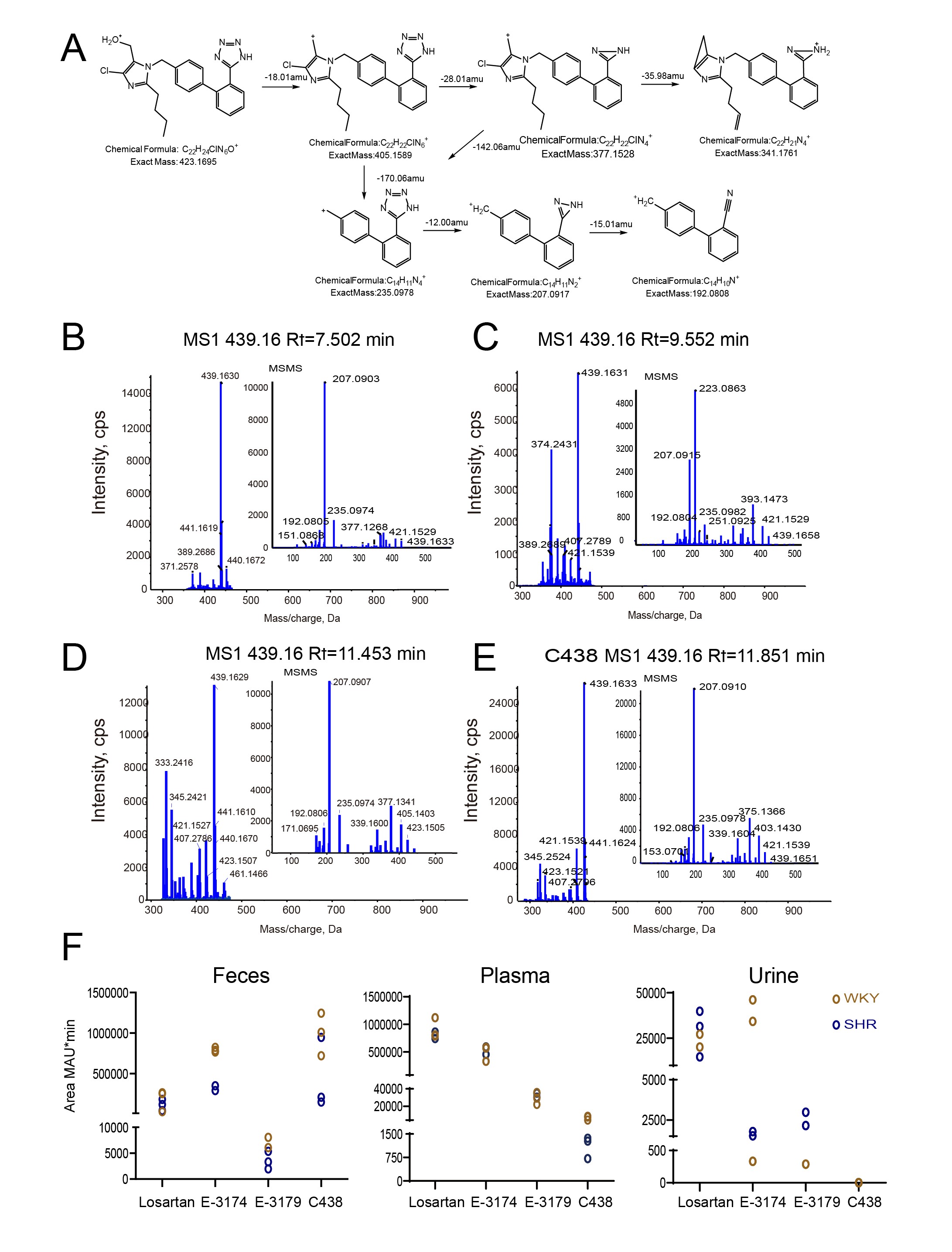


Supplementary Figure 3. Mass spectrometry characteristics and abundance of metabolites of losartan in vivo. (A) Mass spectral fragmentation pattern of losartan. (B-E) Mass spectral fragments of metabolites (m/z 439.16) detected in MS1 and MS2 in vivo. (F) Peak areas of losartan, E-3174, E-3179, and C438 in feces, plasma, and urine mass spectrometry in WKYs and SHRs (n = 3).


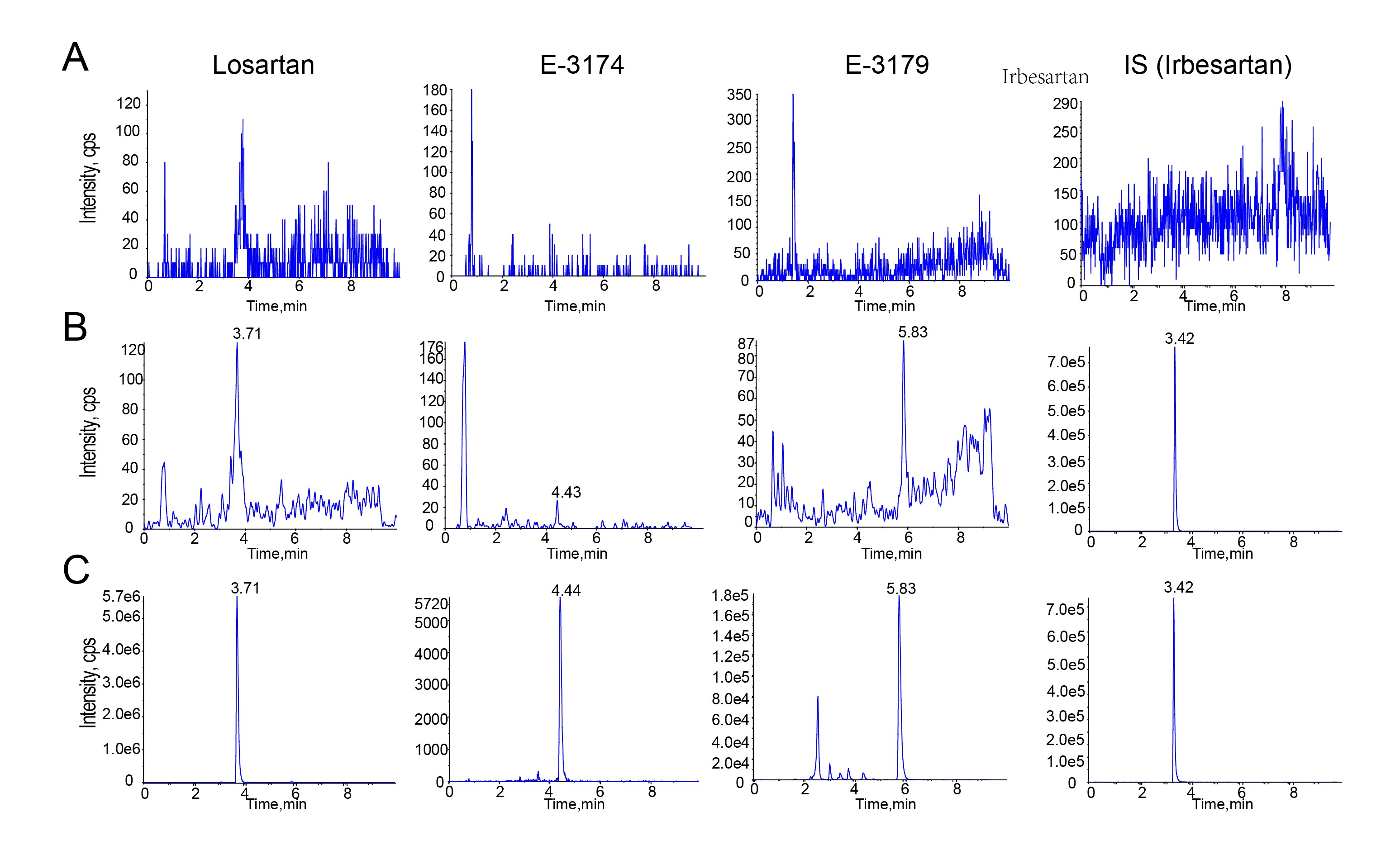
Supplementary Figure 4. Representative multiple reaction monitoring (MRM) chromatograms of losartan, E-3174, E-3179, and IS in rat plasma spiked with IS; (A) blank plasma samples; (B) LLOQ samples of analytes; (C) plasma samples.


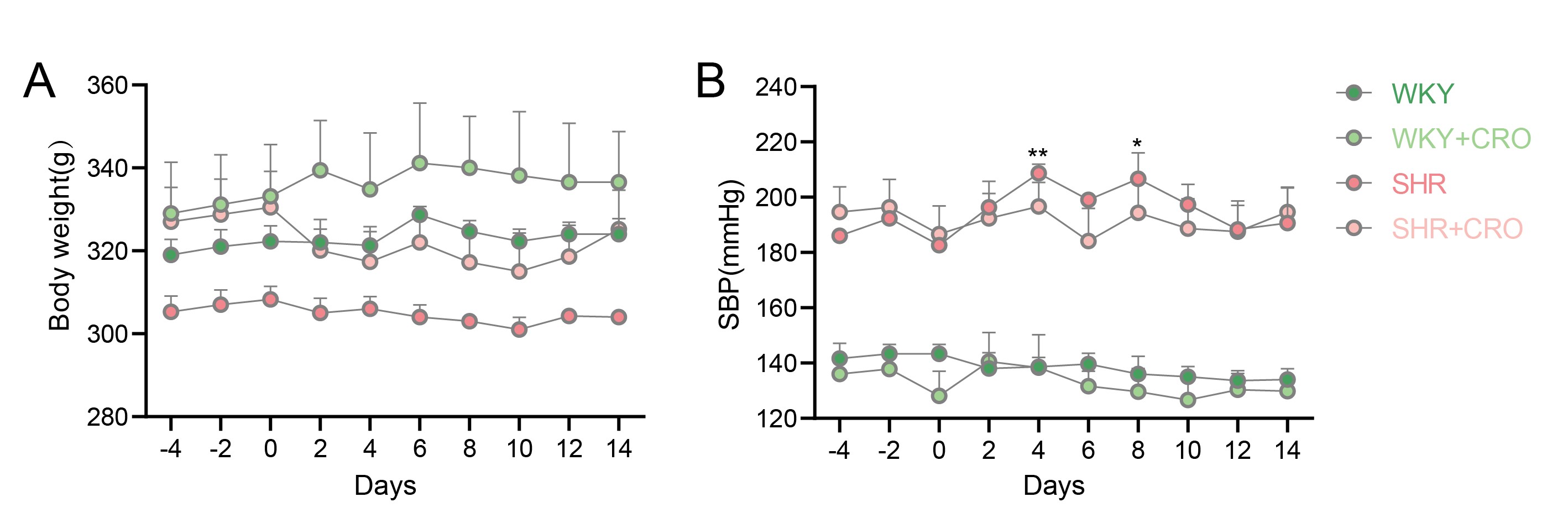
 Supplementary Figure 5. Effect of ceftriaxone sodium on (A) body weight and (B) blood pressure in different groups of WKY and SHR rats (n = 6).


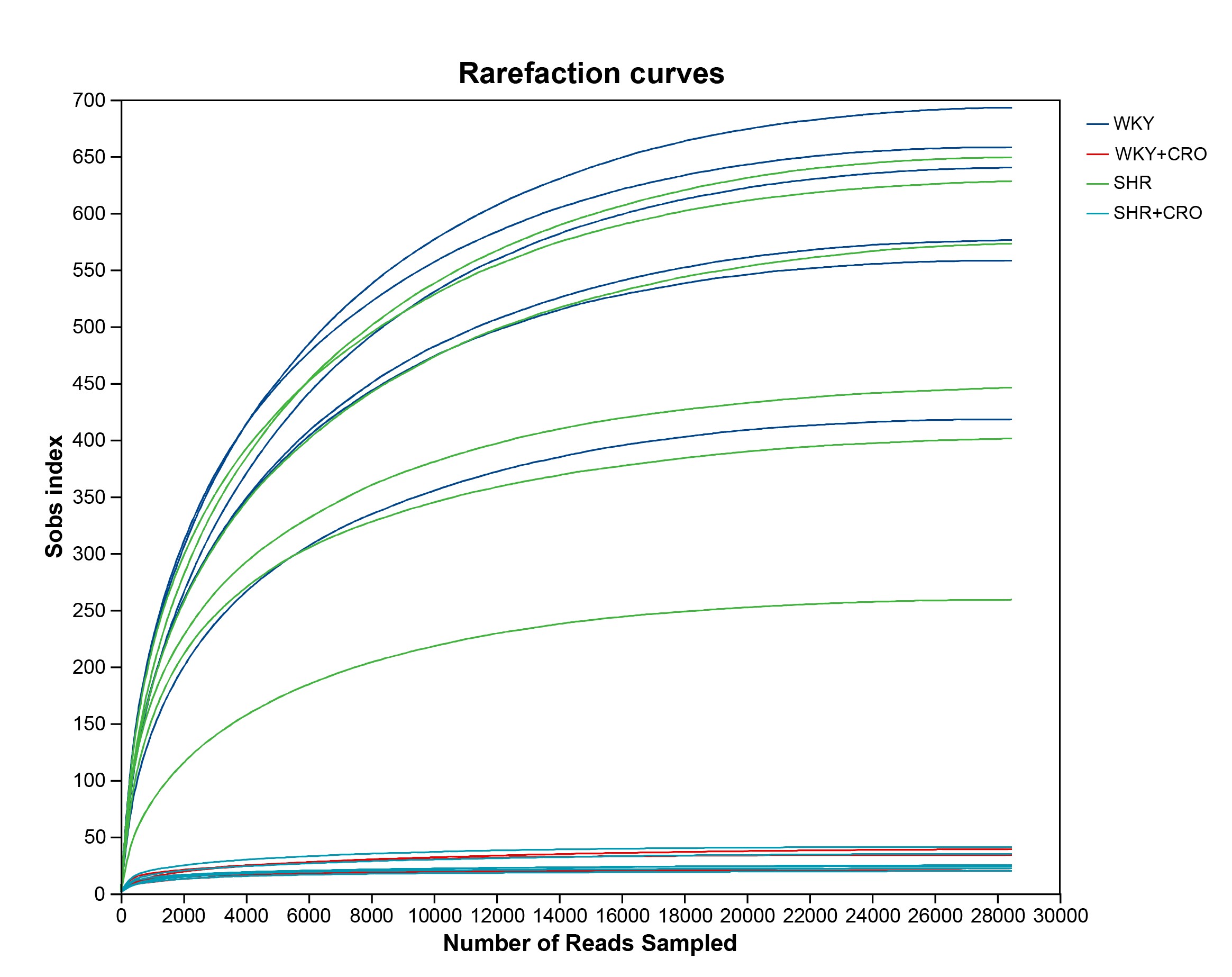


Supplementary Figure 6. Rarefaction curves of the four different groups.


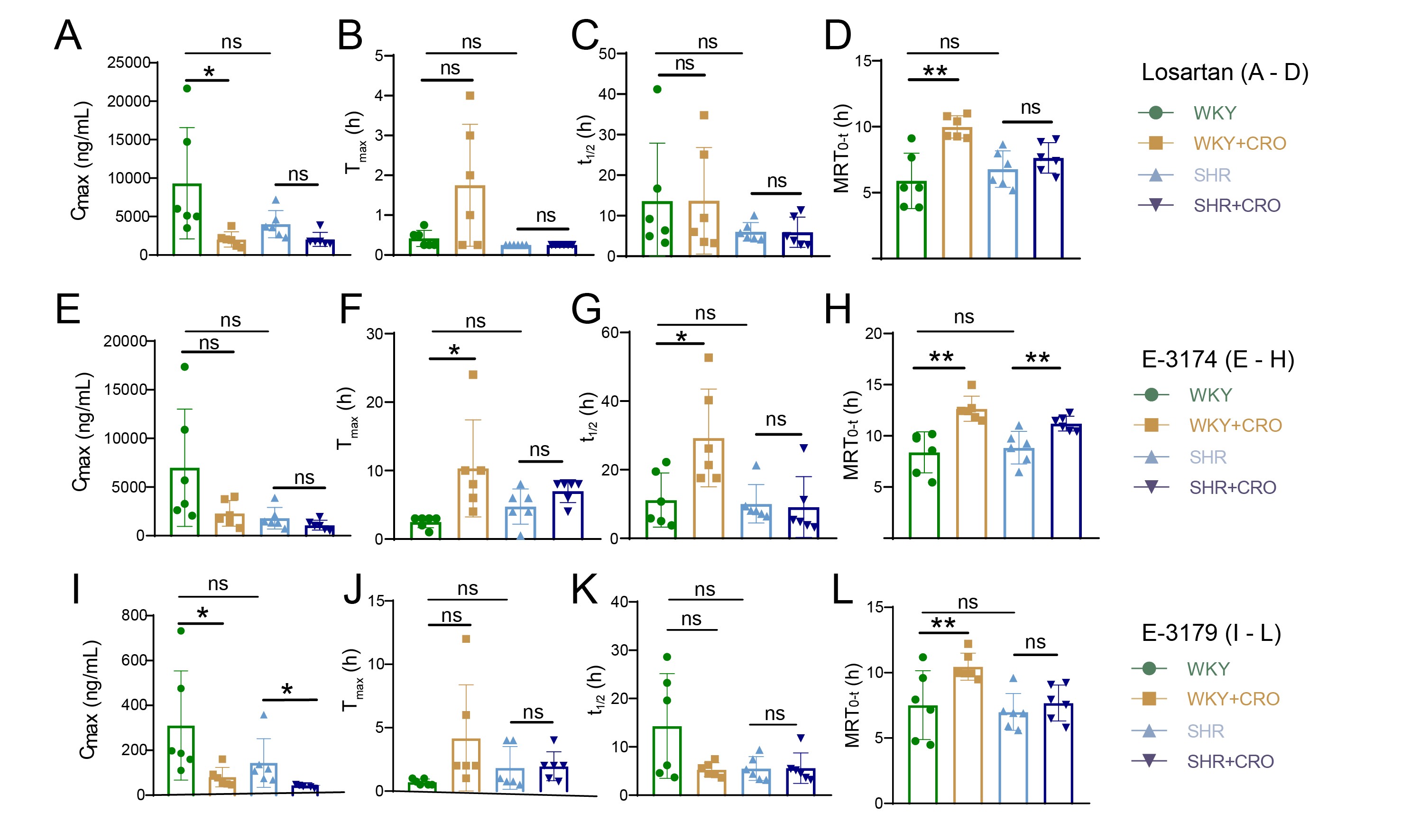
Supplementary Figure 7. Other pharmacokinetic parameters of analytes in different groups: (A-D) losartan; (E-H) E-3174; (I-L) E-3179.


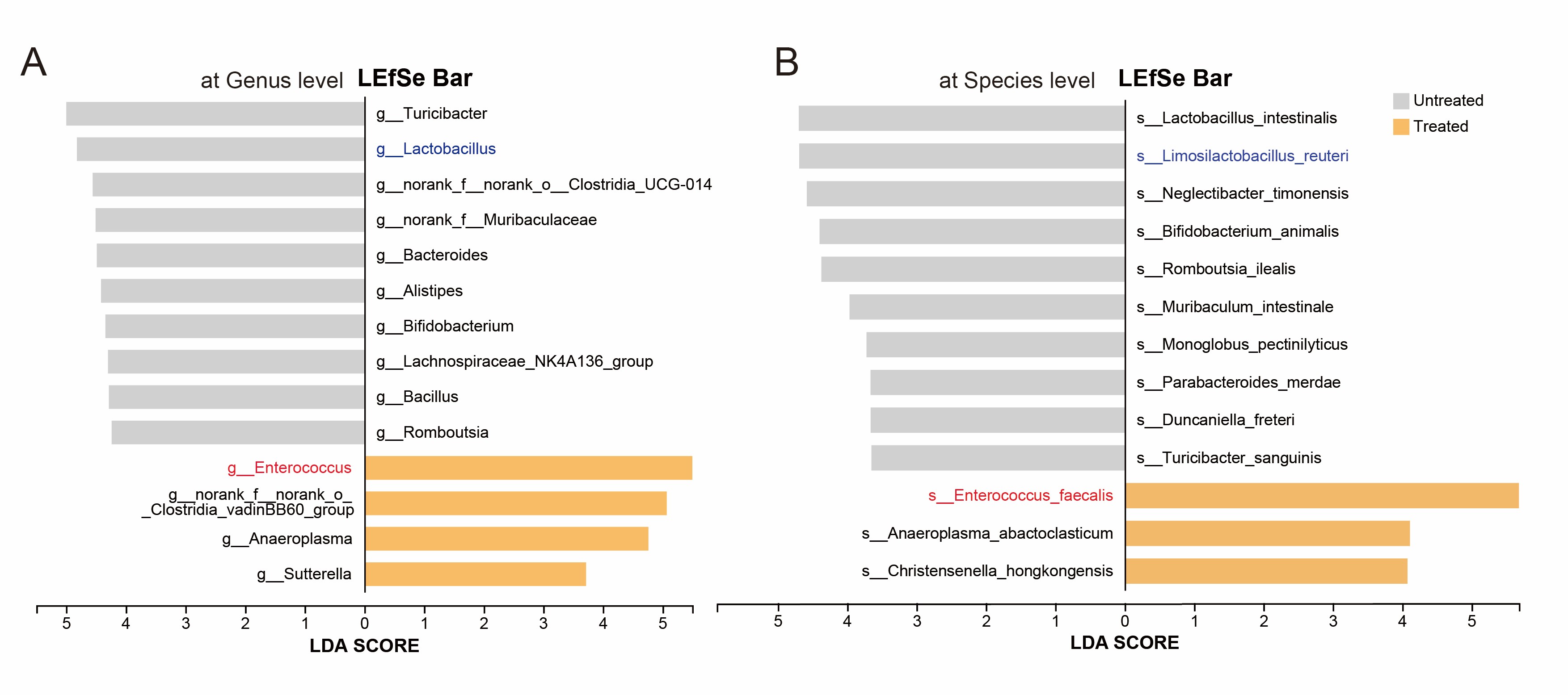


Supplementary Figure 8. Linear Discriminant Analysis (LDA) score of the significantly altered at genus (A) and species (B) levels between treated and untreated CRO groups.


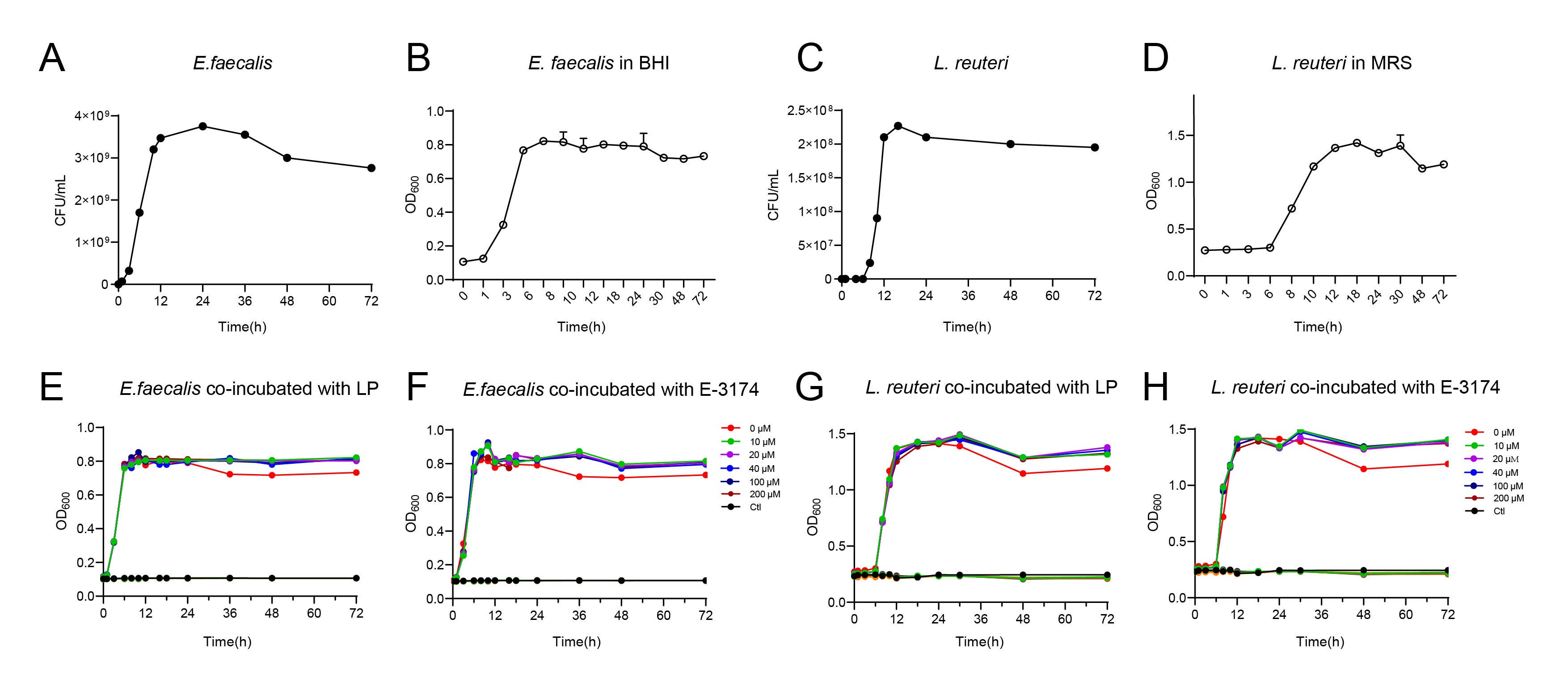


Supplementary Figure 9. Growth characteristics of *E. faecalis* and *L. reuteri*. (A-B) colony-forming unit (CFU) counts and growth curve of *E. faecalis*; (C-D) CFU counts and growth curve of *L. reuteri*; (E-F) Growth curve of *E. faecalis* co-cultured with different concentrations of losartan; (G-H) Growth curve of *L. reuteri* co-cultured with different concentrations of E-3174.


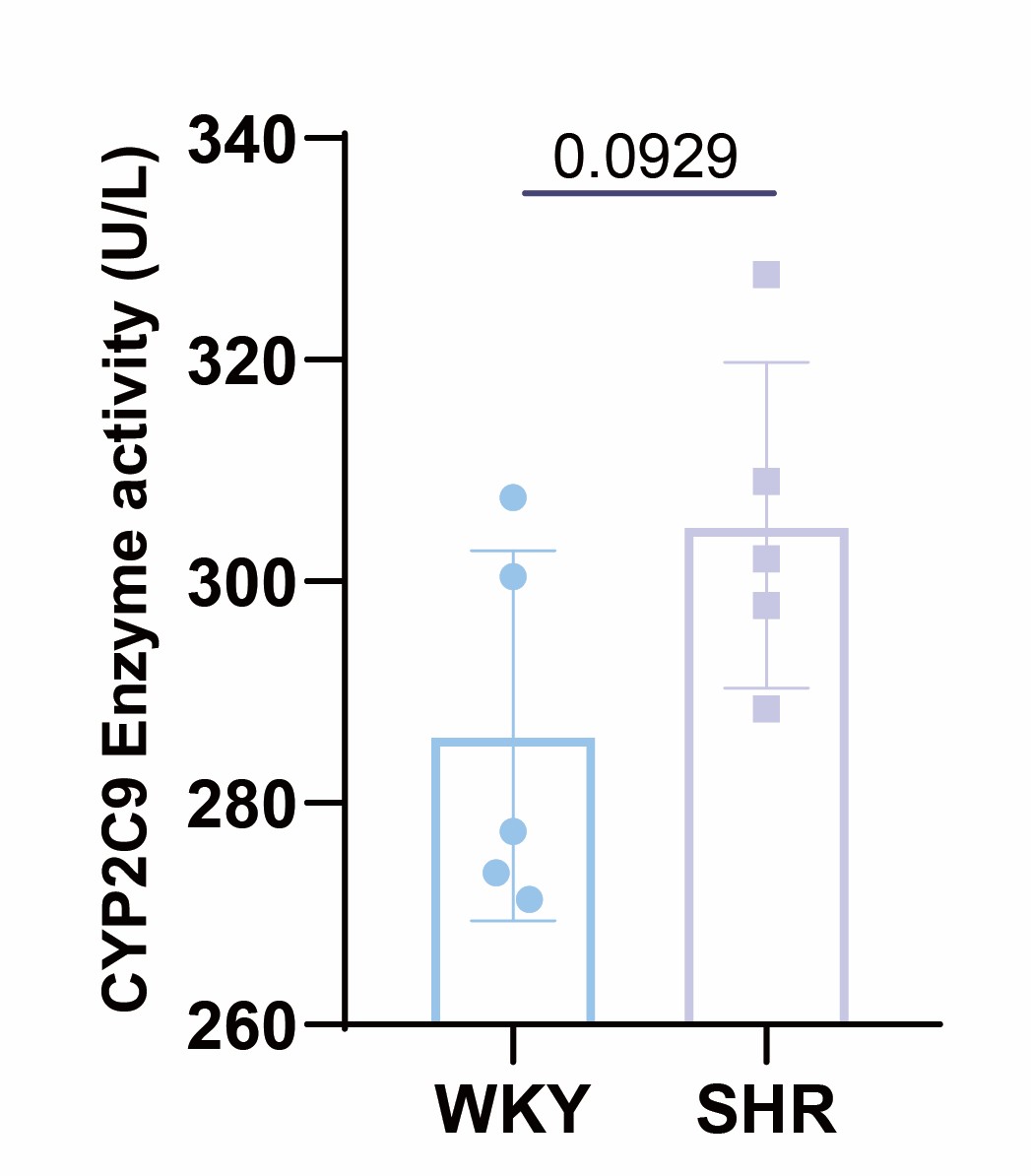


Supplementary Figure 10. Hepatic CYP2C9 enzyme activity in WKY and SHR rats.


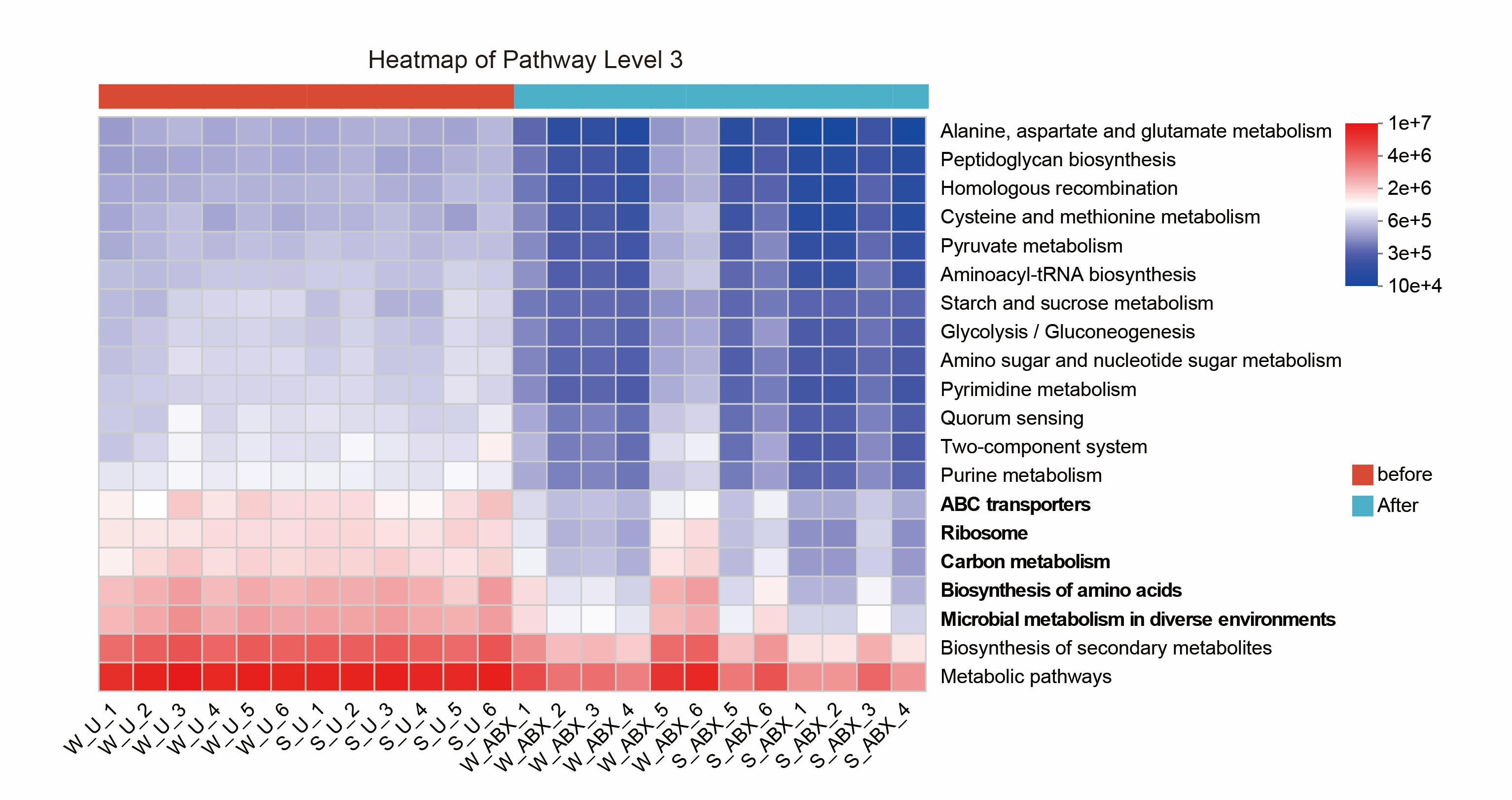


Supplementary Figure 11. Functional prediction of fecal bacterial microbiota by PICRUSt2 combined with the KEGG database, showing Level 3 KEGG pathway results.

- 1. **Supplementary Tables**

Supplementary Table 1. MS parameters and IDA criteria.

| Parameter | ESI^＋^ |
| --- | --- |
| Ion source gas 1 (psi) | 55 |
| Ion source gas 2 (psi) | 55 |
| Ion spray voltage (V) | 5500 |
| Curtain gas (psi) | 35 |
| CAD gas (psi) | 7 |
| Ion source temperature (℃) | 550 |
| Declustering potential ± spread (V) | 80 ± 0 |
| Mass range (Da) | 50-1000 |
| **TOF MS** |  |
| Accumulation time (s) | 0.15 |
| Collision energy ± spread (V) | 10 ± 0 |
| Time bins to sum | 4 |
| **IDA** |  |
| Accumulation time (s) | 0.15 |
| Collision energy ± spread (V) | 35 ± 10 |
| Maximum candidate ions | 10 |
| Intensity threshold (counts/s) | 100 |
| Dynamic background subtraction | True |
| Mass tolerance (mDa) | ± 50 |

Supplementary Table 2. Summary of residual drug content after co-incubation with gut microbiota from WKYs and SHRs.

| Time (h) | Blank medium  mGAM | Remaining content of losartan (μM) | | Remaining content of E-3174 (μM) | |
| --- | --- | --- | --- | --- | --- |
|  |  | WKY | SHR | WKY | SHR |
| 0 | 40.49 ± 1.52 | 37.78 ± 2.05 | 37.62 ± 1.03 | 37.45 ± 1.33 | 38.21 ± 0.11 |
| 12 | 37.44 ± 1.25 | 36.96 ± 2.06 | 38.41 ± 2.14 | 41.40 ± 1.42 | 39.55 ± 2.13 |
| 24 | 37.57 ± 1.21 | 36.03 ± 0.78 | 33.51 ± 2.35 | 39.27 ± 1.11 | 35.95 ± 1.41 |
| 48 | 37.40 ± 1.19 | 33.85 ± 0.96 | 32.98 ± 2.19** | 31.95 ± 1.41 | 27.07 ± 6.32** |

Note: For each time point, WKY and SHR groups were compared to mGAM group. ** *p* < 0.01.

Supplementary Table 3. Calibration curves, correlation coefficient, linear range, and LLOQ of analytes.

| Analytes | Regression  equation | Linearity range  (ng/mL) | Regression  coefficient (r^2^) | LLOQ  (ng/mL) |
| --- | --- | --- | --- | --- |
| Losartan | Y = 9.17E-4 X + 2.33E-4 | 0.1-10000 | 0.9979 | 0.1 |
| E-3174 | Y = 6.14E-6 X - 1.60E-6 | 5.0-5000 | 0.9909 | 5.0 |
| E-3179 | Y = 9.80E-4 X-8.87E-6 | 0.2~2000 | 0.9988 | 0.2 |

Supplementary Table 4. The accuracy, precision, extraction recovery and matrix effect of losartan, E-3174 and E-3179.

| Analyte | Spiked conc. | Intra-day  (mean ± SD, n=6) | | | Inter-day  (mean ± SD, n=18) | | | Extraction  recovery | | Matrix  effect | |
| --- | --- | --- | --- | --- | --- | --- | --- | --- | --- | --- | --- |
|  | (ng/mL) | Found conc.  (ng/mL) | Precision RSD (%) | Accuracy RE (%) | Found conc.  (ng/mL) | Precision RSD (%) | Accuracy RE (%) | Mean  (%) | RSD  (%) | Mean  (%) | RSD  (%) |
| Losartan | LLOQ (0.1) | 0.10 | 6.34 | 2.36 | 0.10 | 7.83 | 2.54 | / | / | / | / |
|  | LQC (0.2) | 0.21 | 8.38 | 2.62 | 0.20 | 7.70 | 2.07 | 101.88 | 6.46 | 102.47 | 7.46 |
|  | MQC (2000) | 2280.96 | 0.62 | 14.05 | 2124.95 | 4.28 | 6.25 | 102.38 | 1.25 | 99.27 | 3.37 |
|  | HQC (8000) | 8019.78 | 1.61 | 0.25 | 8615.15 | 2.42 | 7.69 | 108.68 | 3.41 | 108.92 | 1.87 |
| E-3174 | LLOQ (5) | 4.26 | 4.79 | -14.74 | 5.07 | 10.73 | 1.34 | / | / | / | / |
|  | LQC (10) | 10.03 | 6.45 | 0.32 | 9.66 | 14.28 | -3.40 | 95.52 | 4.26 | 97.55 | 2.31 |
|  | MQC (1000) | 1095.72 | 2.27 | 9.57 | 1025.27 | 7.71 | 2.53 | 106.36 | 1.18 | 102.55 | 4.00 |
|  | HQC (4000) | 4189.71 | 2.62 | 4.74 | 4120.83 | 2.98 | 3.02 | 99.09 | 4.50 | 99.80 | 3.98 |
| E-3179 | LLOQ (0.2) | 0.18 | 7.21 | -10.73 | 0.19 | 8.92 | -3.00 | / | / | / | / |
|  | LQC (0.4) | 0.42 | 4.13 | 6.21 | 0.40 | 13.11 | -0.34 | 102.63 | 5.46 | 105.74 | 2.09 |
|  | MQC (400) | 439.88 | 0.86 | 9.97 | 438.77 | 2.07 | 9.69 | 101.60 | 1.98 | 97.43 | 1.06 |
|  | HQC (1600) | 1726.61 | 2.80 | 7.91 | 1641.15 | 2.15 | 2.57 | 104.66 | 3.44 | 103.37 | 2.84 |

Supplementary Table 5. Stability of the analytes in rat plasma at different storage conditions.

| Analyte | Nominal conc.  (ng/mL) | Autosampler  for 24 h | | Room temperature  for 6 h | | Three freeze-thaw  cycles | | −80°C  for 30 days | |
| --- | --- | --- | --- | --- | --- | --- | --- | --- | --- |
|  |  | RSD (%) | RE (%) | RSD (%) | RE (%) | RSD (%) | RE (%) | RSD (%) | RE (%) |
| Losartan | 0.2 | 3.61 | -4.05 | 1.10 | 12.66 | 4.32 | -2.68 | 6.62 | -7.51 |
|  | 2000 | 0.31 | 12.50 | 1.44 | 10.42 | 2.37 | 9.49 | 0.59 | 12.98 |
|  | 8000 | 0.58 | 8.67 | 1.03 | 5.37 | 0.25 | 6.18 | 0.45 | 4.25 |
| E-3174 | 10 | 0.62 | -4.63 | 4.62 | 3.95 | 4.13 | 0.33 | 4.35 | 1.75 |
|  | 1000 | 1.88 | -1.40 | 2.20 | 11.23 | 1.90 | 8.12 | 2.62 | 5.09 |
|  | 4000 | 1.74 | 2.69 | 1.28 | 0.27 | 1.90 | 5.22 | 2.93 | 5.50 |
| E-3179 | 0.4 | 3.26 | 2.96 | 1.20 | -2.22 | 3.26 | 2.96 | 4.80 | 3.27 |
|  | 400 | 1.37 | 12.05 | 3.14 | 4.87 | 0.66 | 2.84 | 0.34 | 9.16 |
|  | 1600 | 0.64 | 2.34 | 1.74 | -10.69 | 0.55 | 6.66 | 2.82 | 7.58 |

Supplementary Table 6. Dilution integrity of losartan, E-3174, and E-3179.

| Analyte | Spiked conc.  (ng/mL) | Dilution  factor | Found conc.  (ng/mL) | Precision  RSD (%) | Accuracy  RE (%) |
| --- | --- | --- | --- | --- | --- |
| Losartan | 0.2 | 100,000 | 0.20 | 5.28 | −0.54 |
|  | 20 | 1000 | 21.39 | 1.46 | 6.94 |
|  | 2000 | 10 | 2219.98 | 1.26 | 11.00 |
| E−3174 | 10 | 10,000 | 10.42 | 2.39 | 4.24 |
|  | 1000 | 100 | 1074.89 | 3.14 | 7.49 |
|  | 10000 | 10 | 10960.22 | 0.82 | 9.60 |
| E−3179 | 0.4 | 10,000 | 0.38 | 3.65 | −5.08 |
|  | 40 | 100 | 38.79 | 1.61 | −3.03 |
|  | 400 | 10 | 353.83 | 0.15 | −11.54 |

Supplementary Table 7. Pharmacokinetic parameters of losartan, E-3174 and E-3179 in different groups.

| Analyte | Parameter (Unit) | WKY | SHR | WKY+CRO | SHR+CRO |
| --- | --- | --- | --- | --- | --- |
| Losartan | MRT_0-t_ (h) | 5.89±2.10 | 6.79±1.38 | 9.99±0.84 ## | 7.64±1.16 |
|  | MRT _0-∞_ (h) | 16.70±19.24 | 9.65±3.34 | 23.65±16.03 | 12.00±2.70 |
|  | t_1/2z_ (h) | 13.61±14.30 | 6.04±2.28 | 13.67±13.15 | 5.91±3.72 |
|  | T_max_ (h) | 0.42±0.20 | 0.25±0.00 | 1.75±1.53 | 0.25±0.00 |
| E-3174 | MRT_0-t_ (h) | 8.39±2.00 | 8.84±1.59 | 12.64±1.23 ## | 11.21±0.73 ## |
|  | MRT _0-∞_ (h) | 16.93±10.50 | 15.13±8.39 | 48.15±19.94 ## | 21.63±10.07 |
|  | t_1/2z_ (h) | 11.18±7.91 | 10.06±5.59 | 29.27±14.25 # | 9.10±8.85 |
|  | T_max_ (h) | 2.50±0.84 | 4.75±2.56 | 10.33±7.09 # | 7.00±1.67 |
| E-3179 | MRT_0-t_ (h) | 6.18±1.58 | 6.99±1.41 | 10.46±1.03 ## | 7.68±1.38 |
|  | MRT _0-∞_ (h) | 8.79±4.19 | 9.57±2.83 | 15.25±4.02 # | 11.33±2.86 |
|  | t_1/2z_ (h) | 5.75±2.71 | 5.51±2.46 | 5.29±1.44 | 5.61±3.15 |
|  | T_max_ (h) | 0.71±0.25 | 1.83±1.69 | 4.17±4.22 | 1.96±1.15 |

Note: ^#^ represents comparison between WKY and WKY+CRO groups, and also between SHR and SHR+CRO groups, ^##^ *p* < 0.01.

Supplementary Table 8. Differentially abundant gut microbiota species at the species level (LDA>3.5, *p*<0.05) between treated and untreated rats, identified by LEfSe analysis

| Species name | group | Mean | LDA value | P_value |
| --- | --- | --- | --- | --- |
| s__Enterococcus_faecalis | Treated | 5.986722 | 5.681202 | 0.003948 |
| s__Lactobacillus_intestinalis | Untreated | 4.950505 | 4.696167 | 0.0037 |
| s__Limosilactobacillus_reuteri | Untreated | 4.988707 | 4.68986 | 0.002802 |
| s__Neglectibacter_timonensis | Untreated | 4.795187 | 4.581464 | 0.002802 |
| s__Bifidobacterium_animalis | Untreated | 4.642749 | 4.396567 | 0.02223 |
| s__Romboutsia_ilealis | Untreated | 4.747012 | 4.371083 | 0.002802 |
| s__Anaeroplasma_abactoclasticum | Treated | 3.333767 | 4.110518 | 0.007893 |
| s__Christensenella_hongkongensis | Treated | 3.176159 | 4.074756 | 0.013195 |
| s__Muribaculum_intestinale | Untreated | 4.210712 | 3.965158 | 0.007397 |
| s__Monoglobus_pectinilyticus | Untreated | 3.935827 | 3.720299 | 0.002093 |
| s__Parabacteroides_merdae | Untreated | 3.857824 | 3.663514 | 0.007397 |
| s__Duncaniella_freteri | Untreated | 3.898896 | 3.661605 | 0.002093 |
| s__Turicibacter_sanguinis | Untreated | 3.88519 | 3.650142 | 0.002093 |

1. **Supplementary References**

[1] Puris, E., Pasanen, M., Gynther, M., Häkkinen, M. R., Pihlajamäki, J., Keränen, T., et al. (2017). A liquid chromatography-tandem mass spectrometry analysis of nine cytochrome P450 probe drugs and their corresponding metabolites in human serum and urine. Anal Bioanal Chem 409, 251–268. doi: 10.1007/s00216-016-9994-x

[2] Want, E. J., Wilson, I. D., Gika, H., Theodoridis, G., Plumb, R. S., Shockcor, J., et al. (2010). Global metabolic profiling procedures for urine using UPLC-MS. Nat Protoc 5, 1005–1018. doi: 10.1038/nprot.2010.50

[3] Karu, N., Deng, L., Slae, M., Guo, A. C., Sajed, T., Huynh, H., et al. (2018). A review on human fecal metabolomics: Methods, applications and the human fecal metabolome database. Anal Chim Acta 1030, 1–24. doi: 10.1016/j.aca.2018.05.031

[4] Salvadori, M. C., Moreira, R. F., Borges, B. C., Andraus, M. H., Azevedo, C. P., Moreno, R. A., et al. (2009). Simultaneous determination of losartan and hydrochlorothiazide in human plasma by LC/MS/MS with electrospray ionization and its application to pharmacokinetics. Clin Exp Hypertens 31, 415–427. doi: 10.1080/10641960802668714

[5] He, C., Liu, Y., Wang, Y., Tang, J., Tan, Z., Li, X., et al. (2018). 1H NMR based pharmacometabolomics analysis of metabolic phenotype on predicting metabolism characteristics of losartan in healthy volunteers. Journal of Chromatography B 1095, 15–23. doi: 10.1016/j.jchromb.2018.07.016

[6] Wadie, M. A., Kishk, S. M., Darwish, K. M., Mostafa, S. M., and Elgawish, M. S. (2020). Simultaneous Determination of Losartan and Rosuvastatin in Rat Plasma Using Liquid Chromatography–Tandem Mass Spectrometric Technique for Application into Pharmacokinetic and Drug–Drug Interaction Studies. Chromatographia 83, 1477–1494. doi: 10.1007/s10337-020-03967-z

[7] Cusinato, D. A. C., Filgueira, G. C. de O., Rocha, A., Cintra, M. A. C. T., Lanchote, V. L., and Coelho, E. B. (2019). LC-MS/MS analysis of the plasma concentrations of a cocktail of 5 cytochrome P450 and P-glycoprotein probe substrates and their metabolites using subtherapeutic doses. Journal of Pharmaceutical and Biomedical Analysis 164, 430–441. doi: 10.1016/j.jpba.2018.10.029
